# Primary sarcopenia is associated with elevated spontaneous NET formation

**DOI:** 10.3389/fcell.2024.1347495

**Published:** 2024-03-05

**Authors:** Irina Balazs, Manuel Stelzer, Julia Traub, Angela Horvath, Nicole Feldbacher, Vanessa Stadlbauer

**Affiliations:** ^1^ Department of Internal Medicine, Division of Gastroenterology and Hepatology, Medical University of Graz, Graz, Austria; ^2^ Center for Biomarker Research in Medicine (CBmed), Graz, Austria; ^3^ Department of Clinical Medical Nutrition, Medical University of Graz, Graz, Austria

**Keywords:** neutrophils, cirrhosis, sarcopenia, NET formation, chronic inflammation

## Abstract

**Introduction:** Sarcopenia is a frequent complication of liver cirrhosis, but it can also occur independently as a result of any underlying cause. The immune system plays an important role in the pathogenesis of both liver cirrhosis and sarcopenia. Neutrophil function, including neutrophil extracellular trap (NET) formation, is linked to chronic inflammation; however, it has not been extensively studied in patients with sarcopenia. Here, we aim to study if main neutrophil functions, such as phagocytosis, reactive oxygen species (ROS) production, and NET formation, are altered in patients with sarcopenia with or without liver cirrhosis.

**Methods:** Neutrophils from 92 patients (52 patients with liver cirrhosis and sarcopenia, 25 patients with liver cirrhosis without sarcopenia, and 15 patients with sarcopenia without liver cirrhosis) and 10 healthy controls were isolated and stimulated with heat-inactivated *E. coli* (250 bacteria/cell), phorbol 12-myristate 13-acetate (PMA) (100 nM), or incubation medium in duplicates for 2 h at 37°C. Cells were fixed with paraformaldehyde and stained with 4′,6-diamidino-2-phenylindole (DAPI). Pictures of 10 random fields of vision per slide were taken with an Olympus BX51 fluorescence microscope (Olympus, Shinjuku, Tokyo, Japan) at 600x total magnification. The DNA Area and NETosis Analysis (DANA) algorithm was used to quantify the percentage of NET formation per patient. Phagocytosis and ROS production were assessed with the PhagotestTM kit and PhagoburstTM kit (Glycotope, Heidelberg, Germany) in 92 patients and 21 healthy controls, respectively.

**Results:** Spontaneous NET formation was significantly elevated in patients with only sarcopenia compared to patients with cirrhosis and sarcopenia (*p* = 0.008) and healthy controls (*p* = 0.039). NET formation in response to PMA was significantly decreased in patients with cirrhosis (*p* = 0.007), cirrhosis and sarcopenia (*p* < 0.001), and sarcopenia (*p* = 0.002) compared to healthy controls. There was no significant difference in NET formation in response to *E. coli* between the groups. The DANA algorithm was successfully optimized and validated for assessment of clinical samples. There were no significant changes in neutrophil phagocytosis between patients’ groups compared to healthy controls. A significantly lower percentage of neutrophils produced ROS in response to N-formylmethionine-leucyl-phenylalanine (fMLF) in patients compared to healthy controls.

**Discussion:** Spontaneous NET formation might contribute to chronic inflammation and sarcopenia pathogenesis. This, however, does not result in the impairment of the NET formation function of neutrophils in response to a bacterial stimulus and, therefore, cannot be not linked with the increased risk of bacterial infections neither in sarcopenia nor in cirrhosis. The semi-automated NET formation analysis can be successfully implemented to analyze the vast amount of data generated within clinical studies. This approach opens up the possibilities to develop an NET formation-based biomarker in different diseases including sarcopenia and implement NET formation analysis into clinical settings. Phagocytosis and ROS production were not affected in patients with sarcopenia. Further research is needed to explore the mechanism of NET formation in patients with sarcopenia and its potential as a biomarker in sarcopenia.

## 1 Introduction

Sarcopenia is a progressive and generalized skeletal muscle disorder that involves accelerated loss of muscle mass and function, which significantly worsens quality of life. Sarcopenia is associated with a risk of fractures, impairing the ability to perform daily routine activities, and contributes to loss of independence and the need for long-term care placement. Furthermore, it is associated with cardiac and respiratory diseases, cognitive impairment, and increased mortality (Cruz-Jentoft et al., 2019; Cruz-Jentoft and Sayer, 2019). Sarcopenia can occur independently (primary sarcopenia) or can be a complication of various diseases (secondary sarcopenia) including bone and joint diseases, chronic heart failure, chronic obstructive pulmonary disease, diabetes, neurological disorders, cancer, and liver cirrhosis. It is thought that factors such as nutritional imbalances, malabsorption, physical inactivity, age-related changes in skeletal muscles, oxidative stress, motor neuron loss, mitochondrial dysfunction, decreased number and size of myofibers, higher presence of adipose and fibrotic tissues, inflammation, microvascular changes, and apoptosis might contribute to sarcopenia pathogenesis; however, the mechanisms of sarcopenia development are still not fully elucidated (Riuzzi et al., 2018; Cruz-Jentoft and Sayer, 2019; Wang, 2022).

Low-grade chronic systemic inflammation is considered to play an important role in sarcopenia development (Wang, 2022). Neutrophils are the most abundant leukocytes in human blood. They protect the human body primarily against bacteria and fungi and help in defending organisms from parasites and viruses. The main mechanisms of neutrophil-mediated pathogen elimination are phagocytosis, reactive oxygen species (ROS) production, and neutrophil extracellular trap (NET) formation (Ley et al., 2018; Papayannopoulos, 2018). Neutrophils are important players in chronic inflammation development (Herrero-Cervera et al., 2022); however, their function in patients with sarcopenia is barely described.

The existing knowledge gaps result in currently unavailable therapies targeting sarcopenia pathogenesis and uncertain diagnostic criteria (Wilson et al., 2017). Given the high prevalence of sarcopenia (up to 10 percent of people older than 65 years (Anker et al., 2016) and up to 50 percent of people older than 80 years (Wang, 2022)) which tremendously lowers life quality and longevity of people, it is of importance to elucidate the pathogenesis mechanisms of sarcopenia, including the role of the innate immune system, which may aid in developing novel treatment strategies. The aim of this study, therefore, is to study neutrophil phagocytosis, ROS production, and NET formation in patients with sarcopenia with or without liver cirrhosis.

## 2 Materials and methods

### 2.1 Human samples

Patients with clinical/radiological/histological evidence of cirrhosis (*n* = 25), evidence of cirrhosis and sarcopenia (*n* = 52), and of only sarcopenia (*n* = 15) were recruited at the Department of Gastroenterology and Hepatology at the University Hospital of Graz between 2017 and 2019 as part of the study NCT03080129 (ethic vote number: 29-280 ex 16/17). Sarcopenia was diagnosed according to the EWGSOP 2010 criteria. Patients provided written informed consent, and the study was approved by the Medical University of Graz Institutional Review Board and carried out according to the Declaration of Helsinki. Hepatic encephalopathy > grade 2 and/or other cognitive disorders without informed consent, advanced hepatocellular carcinoma (HCC), and age under 18 were exclusion criteria. Patients did not receive steroid treatment (Supplementary Table S1). Healthy controls (*n* = 10) for NET formation assessment were recruited within the same study (NCT03080129). Healthy controls (*n* = 21) for neutrophil phagocytosis and ROS production assessment were recruited within the study NCT01607528 (ethic vote number: 23-096 ex 10/11). All healthy controls provided written informed consent, were older than 18 years, and did not have acute or chronic inflammatory diseases at the moment of blood drawing.

### 2.2 Neutrophil isolation from peripheral blood

Peripheral venous blood was taken in 9-mL VACUETTE^®^ tubes containing lithium heparin (Greiner Bio-One, Kremsmünster, Austria) and carefully placed on an equal amount of Polymorphprep (Axis shield, Oslo, Norway) without mixing the two phases. After centrifugation for 35 min at 500xg, the upper layer of monocytes was carefully discarded with a sterile Pasteur pipette, and the lower layer containing neutrophils was washed with sterile HBSS without Ca^2+^ and Mg^2+^(Thermo Fischer Scientific, Waltham, Massachusetts, United States). Red blood cell lysis buffer (Roche, Basel, Switzerland) was added for 10 min to lyse red blood cells. Cell concentration and viability were counted with the trypan blue exclusion method using a TC20^TM^ Automated Cell Counter.

### 2.3 NET formation assay

The NET formation assay has been described previously (Brinkmann et al., 2010). Isolated neutrophils were resuspended in Roswell Park Memorial Institute (RPMI) 1640 medium supplemented with 2% human serum albumin (CSL Behring, Pennsylvania, United States) and L-glutamine to a concentration of 4 × 105 cells/mL. Then, 500 uL of cells was placed onto sterile round coverslips (13 mm) (VWR, Darmstadt, Germany) inside 24-well plates and incubated for 1 h at 37°C. To each well in duplicates, either 100 μL of 600 nM phorbol 12-myristate 13-acetate (PMA), heat-inactivated *E. coli* BL21 5 × 108 CFU/mL, or RPMI 1640 (negative control) were added, and the plates were incubated for 2 h at 37°C. The cells were then fixed with 4% paraformaldehyde for 15 min, the coverslips were carefully washed with distilled water one time for 5 min, and mounted with ProLong Gold Antifade reagent with 4′,6-diamidino-2-phenylindole (DAPI) (Life Technologies, Carlsbad, United States) on the glass slides. Pictures of 10 random fields of vision per slide were taken with an Olympus BX51 Fluorescence Microscope (Olympus, Shinjuku, Tokyo, Japan) at 600x total magnification. The NET formation percentage was quantified as NET-like structures divided by the total number of neutrophils.

### 2.4 NET formation assay analysis

The DNA Area and NETosis Analysis (DANA) algorithm based on ImageJ/Java was used to quantify the NET formation percentage, as described before (Rebernick et al., 2018), but after optimization and validation for the study samples. ImageJ/Fiji was used to quantify the area, raw integrated density, aspect ratio, roundness, maximum and minimum brightness, and solidity of each region of interest (ROI) (in this context—neutrophil) for each image. Intermodes auto threshold and Phansalkar auto local threshold were used for ROI segmentation. Then, the Java-based part of the DANA algorithm was used for semi-automated quantification of NET percentage per slide providing calculations of area, relative area compared to condensed nuclei and NET status for each ROI, number of NETs, percentage of NET formation, and average cellular DNA for the image. This part of the DANA algorithm was run with the upper elimination cut off of 1.2 and NET cut off value of 4.30. Cell fragments or multiples were automatically eliminated by software based on the optimized standardized cut offs.

### 2.5 Phagocytosis

Peripheral venous blood was taken in VACUETTE^®^ tubes containing 3.8% sodium citrate (Greiner Bio-One, Kremsmünster, Austria). The Phagotest^®^ kit (Glycotope, Heidelberg, Germany) was used to determine the phagocytic capacity of neutrophils according to the manufacturer’s instructions. In brief, 100 μL of whole blood was mixed with 20 μL of stabilized and opsonized FITC-labeled *E. coli* suspension (2 × 109 bacteria/mL) in two tubes. One tube (control) stayed on ice; another one was incubated for 10 min at 37°C. A quenching solution was added to avoid counting of the cell membrane-attached not engulfed bacteria. After washing, the cells were fixed, and red blood cells were lysed. The LSRII flow cytometer (BD Biosciences, San Jose, California, United States) with BD FACS Diva 6.2 software (BD Biosciences, San Jose, California, United States) was used to record 10,000 neutrophils. Further analysis was performed in FlowJo^TM^ v10 software (BD Biosciences, San Jose, California, United States), and the percentage of non-phagocytic neutrophils as well as phagocytic capacity of neutrophils were calculated as described in Horvath et al. (2016) (Supplementary Figure S2). In brief, the phagocytic capacity was calculated as a weighted geometric mean fluorescence intensity (GMFI) of phagocytic neutrophil populations. FITC-negative neutrophils were defined as non-phagocytic neutrophils. The results were normalized to the *E. coli* batch (average of four to five healthy controls per every *E. coli* batch used).

### 2.6 ROS production

Peripheral venous blood was taken in VACUETTE^®^ tubes containing 3.8% sodium citrate (Greiner Bio-One, Kremsmünster, Austria). The Phagoburst^®^ kit (Glycotope, Heidelberg, Germany) was used to determine the percentage of neutrophils that produce ROS in response to different stimuli according to the manufacturer’s instructions. Briefly, 100 μL of the whole blood was mixed with 20 μL of either wash solution (basal ROS production), N-formylmethionine-leucyl-phenylalanine (fMLF), phorbol myristate acetate (PMA), or stabilized and opsonized (non-labeled) *E. coli* (1-2 x 109 bacteria/mL). The tubes were incubated for 10 min at 37°C. The substrate solution containing dihydrorhodamine 123 was added to all the tubes and incubated for 10 min at 37°C. The cells were fixed, and red blood cells were lysed. The LSRII flow cytometer (BD Biosciences, San Jose, California, United States) with BD FACS Diva 6.2 software (BD Biosciences, San Jose, California, United States) was used to record 10,000 neutrophils. Further analysis was performed in FlowJo^TM^ v10 software (BD Biosciences, San Jose, California, United States) (Supplementary Figure S2).

### 2.7 Data analysis

Statistical analysis and graphical visualization of the results were performed with GraphPad PRISM v9 (GraphPad Software, San Diego, CA, United States). D'Agostino and Pearson test and visual assessment of histograms and QQ plots were applied to assess the data distribution. Unpaired *t*-test or the Mann–Whitney test was used to compare two groups of independent samples, and analysis of variance (ANOVA) with Tukey’s multiple comparisons test or Kruskal–Wallis test with Dunn’s test were used to compare more than two groups of independent samples. Fischer’s exact test was used to compare groups of categorical variables. The Bland–Altman test was used to compare two methodologies to analyze NET formation data. Spearman’s correlation coefficient with Benjamini–Hochberg adjustment for multiple tests and partial Spearman’s correlation coefficient were calculated to assess the variables’ relationships. The results are presented as mean ± standard deviation (SD). *p* < 0.05 was considered statistically significant.

## 3 Results

### 3.1 Patients’ characteristics

Patients with cirrhosis (*n* = 25), patients with cirrhosis and sarcopenia (*n* = 52), and patients with sarcopenia (*n* = 15) did not differ significantly in age. However, the age of healthy controls (n = 10), where NET formation was measured, was significantly less than that of patients in other study groups (*p* < 0.001). There were more male patients than female patients in the cirrhosis and sarcopenia groups than in other groups (cirrhosis vs cirrhosis and sarcopenia, *p* = 0.015; sarcopenia vs sarcopenia and cirrhosis, *p* = 0.018; cirrhosis and sarcopenia vs healthy controls (phagocytosis, ROS), *p* = 0.004; and cirrhosis and sarcopenia vs healthy controls (NET), *p* = 0.003). As expected, patients with sarcopenia had lower body mass index (BMI) than patients without sarcopenia (cirrhosis vs cirrhosis and sarcopenia, *p* = 0.016; cirrhosis vs sarcopenia, *p* < 0.001). Neither the severity of cirrhosis (assessed with Child–Pugh grade and MELD score) nor the distribution of cirrhosis etiologies was different between groups. Patients with cirrhosis had a higher Charlson Comorbidity Index (cirrhosis vs sarcopenia, *p* < 0.001; cirrhosis vs cirrhosis and sarcopenia, *p* = 0.001); highest levels of alanine transaminase (ALT) (cirrhosis vs sarcopenia, *p* = 0.037; cirrhosis vs healthy controls (phagocytosis, ROS) *p* < 0.001; cirrhosis and sarcopenia vs healthy controls (phagocytosis, ROS) *p* = 0.002), aspartate transaminase (AST) (cirrhosis and sarcopenia vs sarcopenia *p* < 0.001; cirrhosis vs sarcopenia *p* < 0.001; cirrhosis vs healthy controls (phagocytosis, ROS) *p* < 0.001; cirrhosis and sarcopenia vs healthy controls (phagocytosis, ROS) *p* < 0.001); highest bilirubin (cirrhosis vs healthy controls (phagocytosis, ROS) *p* < 0.001; cirrhosis and sarcopenia vs healthy controls (phagocytosis, ROS) *p* < 0.001; cirrhosis and sarcopenia vs sarcopenia *p* < 0.001; cirrhosis vs sarcopenia *p* = 0.020); highest C-reactive protein (cirrhosis vs healthy controls (phagocytosis, ROS) *p* < 0.001; cirrhosis and sarcopenia vs healthy controls (phagocytosis, ROS) *p* < 0.001, cirrhosis and sarcopenia vs sarcopenia *p* < 0.001; cirrhosis vs sarcopenia *p* = 0.029); and elevated international normalized ratio (cirrhosis vs healthy controls (phagocytosis, ROS) *p* = 0.001; cirrhosis and sarcopenia vs healthy controls (phagocytosis, ROS) *p* < 0.001, cirrhosis and sarcopenia vs sarcopenia *p* < 0.001; cirrhosis vs sarcopenia *p* < 0.001) compared to patients without cirrhosis. The gamma‐glutamyl transferase (GGT) level was higher than the normal range in all patients’ groups, however, with the highest elevation in the sarcopenia group (cirrhosis vs healthy controls (phagocytosis, ROS) *p* < 0.001; cirrhosis and sarcopenia vs healthy controls (phagocytosis, ROS) *p* < 0.001; and cirrhosis and sarcopenia vs sarcopenia *p* < 0.001). Levels of creatinine, leukocyte count, neutrophil count, and monocyte count were not different between the groups. The serum level of albumin and cholesterol, though within the normal range, was significantly lower in patients with cirrhosis than in patients of other groups (cirrhosis vs healthy controls (phagocytosis, ROS) *p* < 0.001; cirrhosis and sarcopenia vs healthy controls (phagocytosis, ROS) *p* < 0.001; cirrhosis and sarcopenia vs sarcopenia *p* < 0.001; and cirrhosis vs sarcopenia *p* < 0.001). Erythrocyte count was slightly lower than normal values (cirrhosis vs healthy controls (phagocytosis, ROS) *p* < 0.001; cirrhosis and sarcopenia vs healthy controls (phagocytosis, ROS) *p* < 0.001; and cirrhosis and sarcopenia vs sarcopenia *p* = 0.003), and hematocrit was decreased in patients with cirrhosis (cirrhosis vs healthy controls (phagocytosis, ROS) *p* = 0.001; cirrhosis and sarcopenia vs healthy controls (phagocytosis, ROS) *p* < 0.001; and cirrhosis and sarcopenia vs sarcopenia *p* = 0.001). Lymphocyte count of all groups was within the normal range, but the sarcopenia group had the highest values (cirrhosis vs sarcopenia *p* = 0.004; cirrhosis and sarcopenia vs healthy controls (phagocytosis, ROS) *p* = 0.003; cirrhosis and sarcopenia vs sarcopenia *p* < 0.001). Approximately half of the patients with cirrhosis had hepatocellular carcinoma (HCC). All patients’ characteristics are presented in Table 1.

**TABLE 1:** Patients’ characteristics depending on the group.

	Cirrhosis	Cirrhosis and sarcopenia	Sarcopenia	Healthy controls (NET)	Healthy controls (phagocytosis, ROS)	*p*-value
**N**	25	52	15	10	21	
**Age, years**	61 (13)	62 (12)	62 (11)	29 (6)	58 (7)	<0.001*
**Male/female, N**	13/12	42/10	7/8	3/7	9/12	0.015^$^
						0.018^#^
						0.004^&^
						0.003^%^
**BMI**	29.63 (5.05)	25.82 (4.11)	22.7 (3.81)	-	-	0.016^$^
						<0.001^@^
**Child–Pugh grade, A/B/C, N**	14/8/3	22/19/9	-	-	-	ns
**MELD**	11.88 (5.22)	12.73 (4.94)	-	-	-	ns
**Etiology, HCV/alcohol/NASH/other, N**	6/11/5/3	7/33/8/4	-	-	-	ns
**Charlson Comorbidity Index**	5.36 (1.66)	4.65 (1.56)	2.07 (2.92)	-	-	<0.001^@^
						0.001^#^
**HCC, yes/no, N**	15/10	23/29	0/15	0/10	0/21	<0.001^@,a, &^
						0.001^#^
						0.001^
						0.01^%^
**ALT, U/l**	67.12 (91.16)	42.13 (48.04)	27.8 (18.55)	-	20 (5.82)	0.037^@^
						<0.001^a^
						0.002^&^
**AST, U/l**	107.9 (176.9)	70.16 (81.56)	27.27 (16.84)	-	24 (8.79)	<0.001^@,a,#,&^
**GGT, U/l**	164.2 (153)	199.2 (163.1)	262.2 (721.7)	-	20.9 (10.33)	0.032^#^
						<0.001^a,&^
**AP, U/l**	120.1 (58.27)	140.8 (53.49)	94.27 (76.36)	-	62.24 (14.58)	<0.001^a,&,#^
**Bilirubin, mg/dl**	1.99 (2.53)	3.48 (5.74)	0.61 (0.23)	-	0.55 (0.24)	<0.001^a,&,#^
						0.020^@^
**Creatinine, mg/dl**	1.05 (0.49)	1.02 (0.64)	0.86 (0.16)	-	0.91 (0.17)	ns
**Albumin, g/dl**	3.56 (0.53)	3.43 (0.52)	4.39 (0.35)	-	4.18 (0.87)	<0.001^a,&,#, @^
**CRP, mg/l**	11.45 (14.43)	12.00 (12.17)	6.76 (17.35)	-	2.06 (2.19)	<0.001^a,&,#^
						0.029^@^
**Cholesterol, mg/dl**	137.00 (43.63)	135.40 (37.29)	199.70 (37.60)	-	215.50 (30.58)	<0.001^a,&,#, @^
**Neutrophils, x10^9^/l**	4.04 (3.18)	4.50 (2.94)	4.36 (1.37)	-	3.40 (1.52)	ns
**Monocytes, x10^9^/l**	0.53 (0.41)	0.45 (0.19)	0.47 (0.22)	-	0.44 (0.14)	ns
**Leukocytes, x10^9^/l**	6.06 (3.84)	6.28 (3.16)	7.17 (1.84)	-	5.59 (1.78)	ns
**Erythrocytes, x10^6^/l**	3.97 (0.65)	3.75 (0.74)	4.55 (0.46)	-	4.85 (0.43)	<0.001^a,&^
						0.003^#^
**Lymphocytes, x10^9^/l**	1.31 (0.61)	1.16 (0.52)	2.05 (0.66)	-	1.63 (0.47)	0.004^@^
						0.003^&^
						<0.001^#^
**HCT, %**	35.64 (5.88)	33.96 (5.69)	40.73 (3.97)	-	41.83 (3.36)	0.001^a,#^
						<0.001^&^
**INR, ratio**	1.27 (0.28)	1.31 (0.37)	0.94 (0.11)	-	1.00 (0.04)	0.001^a^
						<0.001^&,#, @^

Values are presented as mean (SD), unless stated otherwise. *The healthy controls (NET) group is different from other patients’ groups. $ The cirrhosis group is different from the cirrhosis and sarcopenia group. #Sarcopenia is different from the cirrhosis and sarcopenia group. & The cirrhosis and sarcopenia group is different from healthy controls (phagocytosis, ROS) group. % The cirrhosis and sarcopenia group is different from the healthy controls (NET) group. @The cirrhosis group is different from the sarcopenia group. ^a^Cirrhosis is different from Healthy controls group (Phagocytosis, ROS).

### 3.2 NET formation is dysregulated in patients with sarcopenia

**FIGURE 1:**
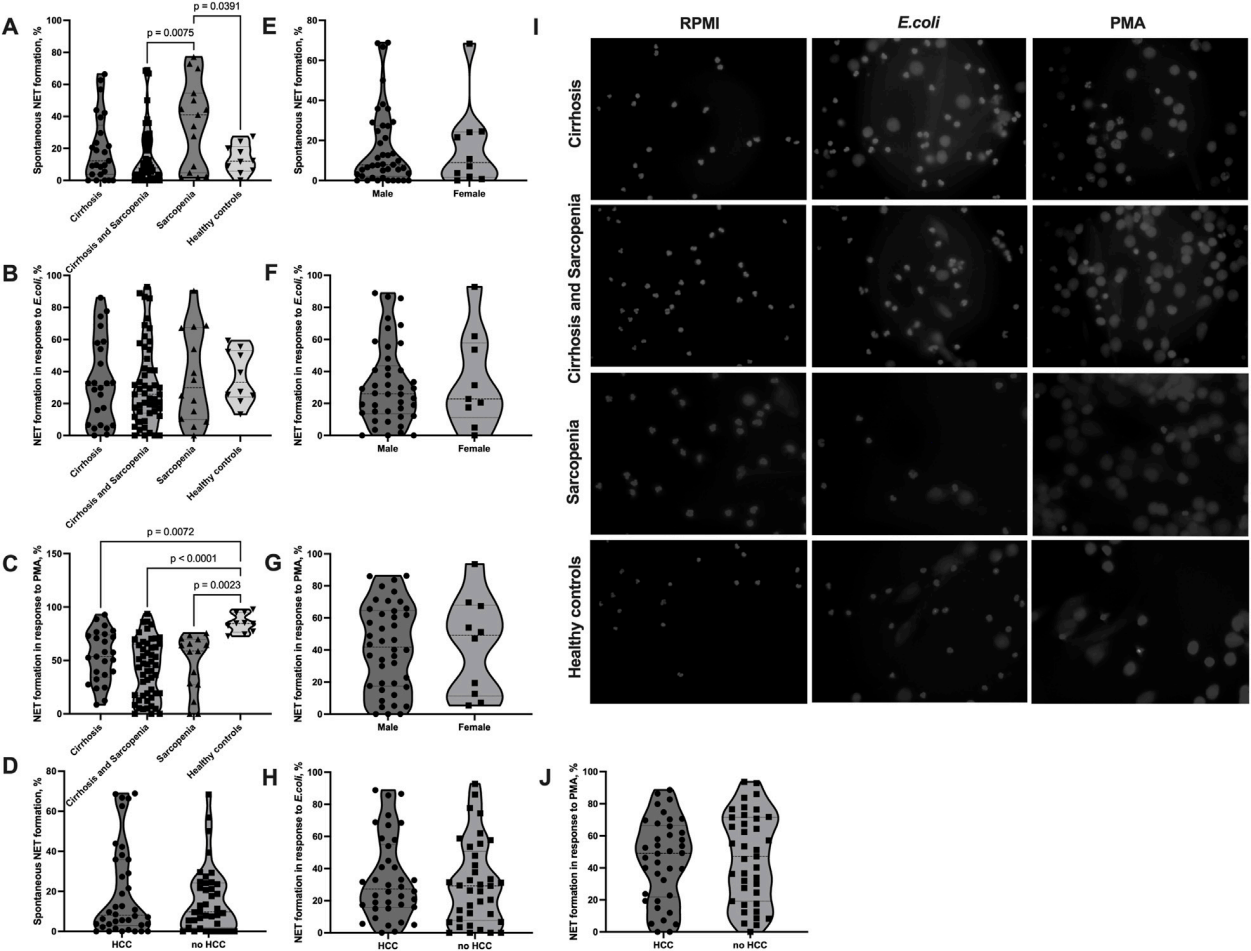
Elevated spontaneous NET formation and decreased NET formation in response to PMA in sarcopenia. **(A–J)** Neutrophils were isolated from the peripheral blood of patients and healthy controls, NET formation was induced with RPMI (spontaneous NET formation) **(A, D, E, I)**, *E.coli* (100 uL of heat-inactivated *E. coli* BL21 5 × 108 CFU/mL per 500 uL of 4 × 105 neutrophils/mL) **(B, F, H, I)**, or 100 nM PMA **(C, G, I, J)** for 2 h at 37°C. The cells were fixed and stained with DAPI. Cell images taken with a fluorescent microscope were analyzed using the DANA algorithm. The NET formation percentage was quantified as NET-like structures divided by the total number of neutrophils. **(A)** Spontaneous NET formation (% of neutrophils producing NET) in cirrhosis patients (*n* = 25), cirrhosis and sarcopenia patients (*n* = 52), sarcopenia patients (*n* = 15), and healthy controls (*n* = 10). **(B)** NET formation in response to *E.coli* (% of neutrophils producing NET) in cirrhosis patients (*n* = 24), cirrhosis and sarcopenia patients (*n* = 51), sarcopenia patients (*n* = 14), and healthy controls (*n* = 10). **(C)** NET formation in response to PMA (% of neutrophils producing NET) in cirrhosis patients (*n* = 25), cirrhosis and sarcopenia patients (*n* = 52), sarcopenia patients (*n* = 15), and healthy controls (*n* = 10). **(D)** Spontaneous NET formation (% of neutrophils producing NET) in patients with HCC (*n* = 38) and patients without HCC (*n* = 39). **(E)** Spontaneous NET formation (% of neutrophils producing NET) in male (*n* = 42) and female (*n* = 10) patients from the cirrhosis and sarcopenia group. **(F)** NET formation in response to *E. coli* (% of neutrophils producing NET) in male (*n* = 42) and female (**n** = 9) patients from the cirrhosis and sarcopenia group. **(G)** NET formation in response to PMA (% of neutrophils producing NET) in male (*n* = 42) and female (*n* = 10) patients from the cirrhosis and sarcopenia group. **(H)** NET formation in response to *E. coli* (% of neutrophils producing NET) in patients with HCC (n = 38) and patients without HCC (*n* = 37) **(I)** Representative pictures of NET formation in neutrophils from patients’ groups and healthy controls after staining with DAPI. **(J)** NET formation in response to PMA (% of neutrophils producing NET) in patients with HCC (*n* = 38) and patients without HCC (*n* = 39). Truncated violin plots show the frequency distribution of measured parameters; the broken line indicates median, and dotted lines indicate quartiles; one-way analysis of variance (ANOVA) with Tukey’s multiple comparisons test **(A–C)**; Mann–Whitney *U* test **(D, E)**; unpaired *t*-test **(F, G, H, J)**. NET: neutrophil extracellular trap; DANA: DNA Area and NETosis Analysis; PMA: phorbol 12-myristate 13-acetate; RPMI: Roswell Park Memorial Institute; DAPI: 4′,6-diamidino-2-phenylindole; HCC: hepatocellular carcinoma.

Our analysis of NET formation revealed dysregulation of neutrophil function in patients with sarcopenia and cirrhosis (Figures 1A–J). All patients’ groups tended to have elevated spontaneous NET formation levels compared to healthy controls, with the highest spontaneous NET formation in patients with sarcopenia without cirrhosis (cirrhosis mean (SD) = 20.21% (20.55), cirrhosis and sarcopenia mean (SD) = 16.20% (19.36), sarcopenia mean (SD) = 35.71% (26.93), and healthy controls mean (SD) = 13.34% (8.89)), which was significantly higher than that in the cirrhosis and sarcopenia group (*p* = 0.008) and healthy controls group (*p* = 0.039) (Figure 1A). On the other hand, all patients’ groups had lower NET formation in response to stimulation with PMA than healthy controls (cirrhosis mean (SD) = 53.67% (23.94), cirrhosis and sarcopenia mean (SD) = 41.85% (27.17), sarcopenia mean (SD) = 47.13% (27.13), healthy controls mean (SD) = 84.84% (8.75), cirrhosis vs healthy controls *p* = 0.007, cirrhosis and sarcopenia vs healthy controls *p* < 0.001, and sarcopenia vs healthy controls *p* = 0.002), with the sarcopenia group having almost no patients, whose neutrophils could mount adequate NET formation in response to PMA (the highest level of NET formation in this’ group of patients was 75.55%) (Figure 1C). NET formation in response to *E. coli* was not different between the groups (cirrhosis mean (SD) = 33.34% (26.49), cirrhosis and sarcopenia mean (SD) = 32.05% (25.47), sarcopenia mean (SD) = 36.38% (28.74), and healthy controls mean (SD) = 36.89% (16.39)) (Figure 1B). Representative images of NET formation are shown in Figure 1I and Supplementary Figure S3. As the cirrhosis and sarcopenia group had a significantly higher number of male patients, we also assessed if NET formation is different between male and female patients within this group to ensure that the differences we saw between this group and other groups cannot be explained by different patients’ characteristics. However, male and female patients had comparable levels of NET formation (Figures 1E–G). NET formation in patients with HCC compared to patients without HCC was not different (Figures 1D, H, J). Since healthy controls were significantly younger than patients (Table 1), we performed correlation analyses between spontaneous NET formation and age of patients (*n* = 92, r = 0.090, *p* = 0.391), NET formation in response to *E. coli* and age of patients (*n* = 89, r = −0.020, *p* = 0.852), and NET formation in response to PMA and age of patients (*n* = 92, r = −0.182, *p* = 0.083), which were not significant. We also assessed whether NET formation correlated with any of clinical parameters of the patients (Supplementary Figure S1). Only a very low positive correlation between NET formation in response to PMA and erythrocyte count (*n* = 89, r = −0.237, *p* = 0.026) and a very low negative correlation between NET formation in response to *E. coli* and INR (*n* = 89, r = −0.240, *p* = 0.025) were significant.

### 3.3 Validation and optimization of the semi-automated NET formation analysis approach

**FIGURE 2:**
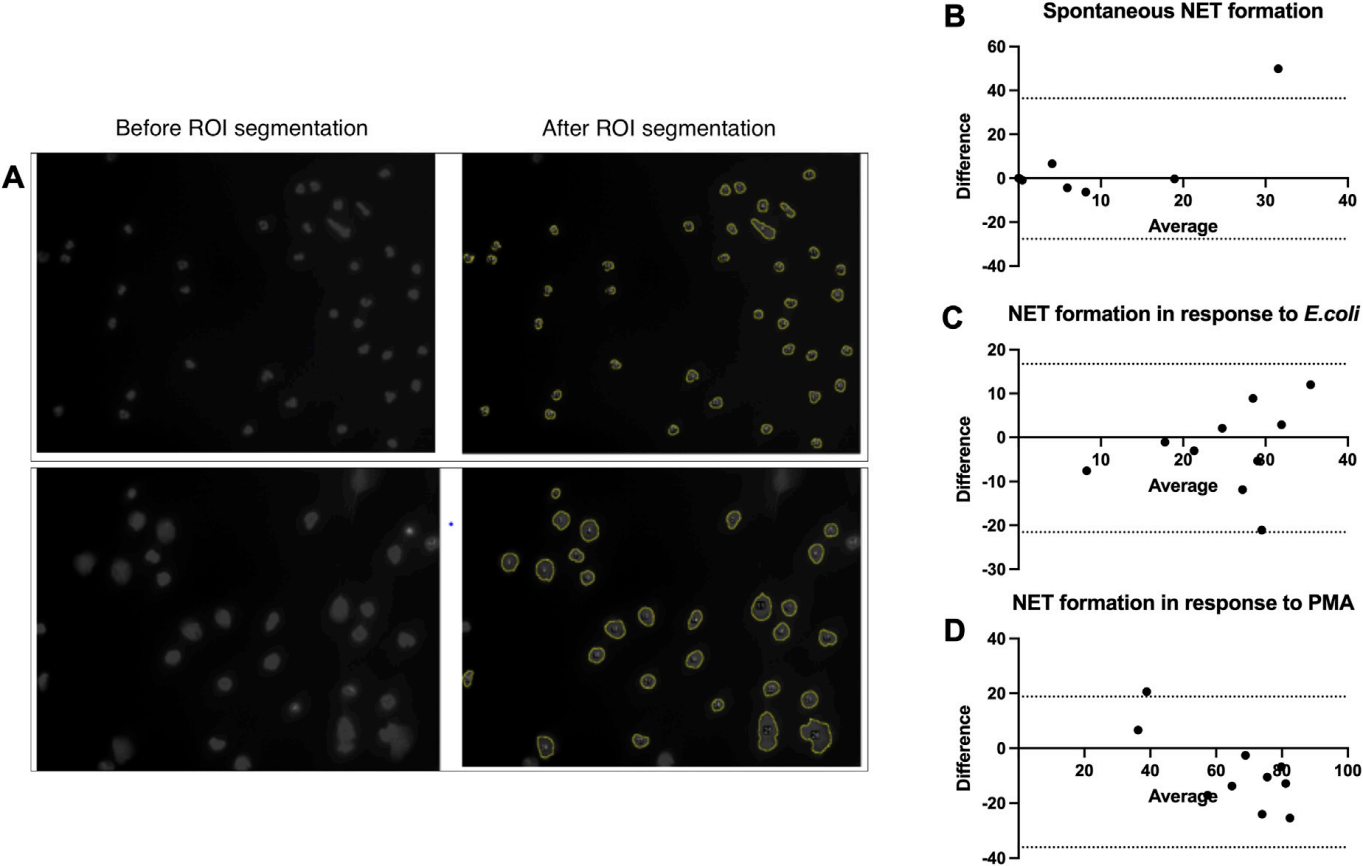
Validation and optimization of the DANA algorithm. **(A–D)** Neutrophils were isolated from peripheral blood of patients and healthy controls, NET formation was induced with RPMI (spontaneous NET formation) **(B)**, *E.coli* (250 bacteria/cell) **(C)**, or 100 nM PMA **(D)** for 2 h at 37°C. The cells were fixed and stained with DAPI. Cell images taken with a fluorescent microscope were analyzed using the DANA algorithm and by eye. The NET formation percentage was quantified as NET-like structures divided by the total number of neutrophils. **(A)** Examples of images before and after ROI segmentation by DANA ImageJ/Fiji plugin. **(B)** Bland–Altman test to compare counting of spontaneous NET formation by eye and DANA in the same samples (*n* = 10), bias (SD) = 4.38 (16.33), 95% limits of agreement = −27.62; 36.39. **(C)** Bland–Altman test to compare counting of NET formation in response to *E.coli* by eye and DANA in the same samples (*n* = 10), bias (SD) = -2.4 (9.77), 95% limits of agreement = −21.54; 16.74. **(D)** Bland–Altman test to compare counting of NET formation in response to PMA by eye and DANA in the same samples (*n* = 10), bias (SD) = -8.59 (14.01), 95% limits of agreement = −36.05; 18.87. DANA: DNA Area and NETosis Analysis; PMA: phorbol 12-myristate 13-acetate; RPMI: Roswell Park Memorial Institute; NET: neutrophil extracellular trap; ROI: region of interest; SD: standard deviation.

NET formation assessment within clinical studies is currently limited by laborious and time-consuming methodologies; especially, quantification of NETs in the microscopy pictures takes a vast amount of time and is prone to bias introduced by the observer. Alternative methods, such as the enzyme-linked immunosorbent assay (ELISA) or fluorometry-based approaches, lack specificity. To overcome this limitation, we optimized the DANA algorithm (Rebernick et al., 2018) of semi-automated NET microscopy picture analysis for the fluorescence microscopy pictures of NET formation we collected in our study. Before we applied the algorithm to our data, we optimized the ImageJ/Fiji plugin and Java-based algorithm for our images and compared its performance to that of the conventional image analysis by the eye with pictures from 10 patients for each condition (spontaneous NET formations, NET formation in response to *E.coli*, and NET formation in response to PMA) (Figures 2A–D). The Bland–Altman test did not show any relevant systematic bias in spontaneous NET formation and NET formation in response to *E. coli* (spontaneous NET formation bias (SD) = 4.38 (16.33); NET formation in response to *E. coli* bias (SD) = -2.4 (9.77)); however, it showed a trend to slight underestimation of NET formation by DANA when analyzing pictures of NET formation in response to PMA (bias (SD) = -8.59 (14.01)) (Figures 2B–D). Given that and the two outliers detected (one outlier was in quantifications of spontaneous NET formation, and another outlier was in quantifications of NET formation in response to PMA) (Figures 2B–D), we applied a quality control when analyzing the samples using DANA. To detect and eliminate possible bias introduced by DANA analysis, we visually reassessed those samples, which had NET formation outside the mean+/-2 standard deviations within the same condition (spontaneous, *E.coli*, and PMA) and patient group to prove that it is a true outlier and not the result of DANA misidentification of cells undergoing NET formation. In case of misinterpretation by DANA, the percentage of NET formation counted visually was taken into further analysis for those samples. Furthermore, we calculated how much time is saved when the semi-automated algorithm is used for the analysis. The use of DANA allowed decreasing the time of analysis from 30 to 5 min per patient on average.

### 3.4 Neutrophil phagocytosis and ROS production are not affected by sarcopenia

**FIGURE 3:**
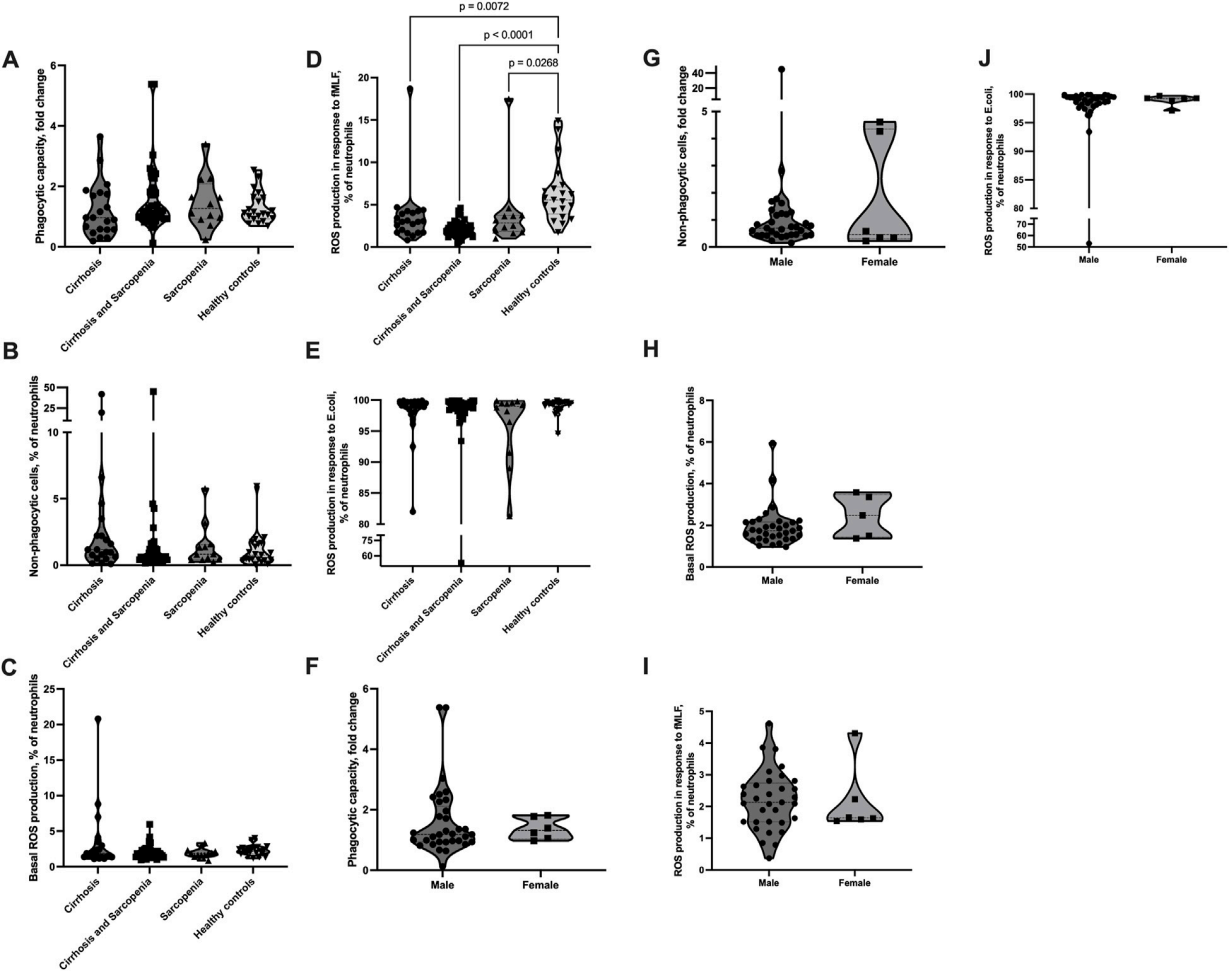
Neutrophil phagocytosis and ROS production are not affected in sarcopenia. **(A–J)** Peripheral venous blood from patients and healthy controls was collected, and the Phagotest^®^ kit and Phagoburst^®^ kit (Glycotope, Heidelberg, Germany) were used to assess neutrophil phagocytosis and ROS production, according to the manufacturer’s instructions. **(A, B, F, G)** Phagocytosis of *E. coli* (4 × 107 bacteria/100 µL blood). **(A, F)** Neutrophil phagocytic capacity normalized to the *E. coli* batch. **(B, G)** Percentage of non-phagocytic neutrophils. **(C–E, H–J)** ROS production. Percentage of neutrophils, which produced ROS **(C, H)** without any stimulus or after 10 min of **(D, I)** fMLF (0.8 µM) or **(E, J)** *E. coli* (2-4x107 bacteria/100 µL blood) stimulation. **(A–E)** Cirrhosis *n* = 21, cirrhosis and sarcopenia *n* = 39, sarcopenia *n* = 12, healthy controls n = 21; **(F–I)** cirrhosis and sarcopenia group male *n* = 33, female = 6. Truncated violin plots show the frequency distribution of measured parameters; the broken line indicates median, and dotted lines indicate quartiles; Kruskal–Wallis test with Dunn’s test **(A–E)**; Mann–Whitney *U* test **(F–J)**. ROS: reactive oxygen species; fMLF: N-formyl-met-leu-phe.

The phagocytic capacity of neutrophils and number of non-phagocytic neutrophils were not significantly different between patients’ groups and between patients and healthy controls; however, patients from the cirrhosis group tended to have a lower phagocytic capacity and higher number of non-phagocytic cells (phagocytic capacity: cirrhosis mean (SD) = 1.21 (0.89), cirrhosis and sarcopenia mean (SD) = 1.57 (1.09), sarcopenia mean (SD) = 1.47 (0.85), healthy controls mean (SD) = 1.29 (0.51); non-phagocytic cells: cirrhosis mean (SD) = 4.39 (9.59), cirrhosis and sarcopenia mean (SD) = 2.11 (7.12), sarcopenia mean (SD) = 1.41 (1.6), and healthy controls mean (SD) = 1.09 (1.28)) (Figures 3A, B). The number of neutrophils with basal ROS production and ROS production in response to *E. coli* was also not significantly different between the groups (Figures 3C, E). The number of neutrophils with ROS production in response to fMLF was slightly but significantly higher in the healthy controls group than in the patients’ groups (cirrhosis mean (SD) = 3.62 (3.63), cirrhosis and sarcopenia mean (SD) = 2.19 (0.94), sarcopenia mean (SD) = 3.97 (4.39), and healthy controls mean (SD) = 6.29 (3.46); cirrhosis vs healthy controls *p* = 0.007, cirrhosis and sarcopenia vs healthy controls *p* < 0.001, and sarcopenia vs healthy controls *p* = 0.027) (Figure 3D). As the “cirrhosis and sarcopenia” group had a significantly higher number of male patients, we also assessed if neutrophil phagocytosis and ROS production parameters are different between male and female patients within this group. Male and female patients had comparable levels of neutrophil phagocytosis and ROS production (Figures 3F–J).

## 4 Discussion

Sarcopenia and liver cirrhosis are associated with chronic inflammation and alterations in the immune system (Cruz-Jentoft and Sayer, 2019; Wang, 2022; Balazs and Stadlbauer, 2023); however, the role of NET formation in their pathogenesis is not well-described yet. Here, we aimed to study NET formation and other basic functions of neutrophils, such as phagocytosis and ROS production, in patients with sarcopenia, cirrhosis, and cirrhosis-associated sarcopenia.

NET formation is a relatively newly discovered function of neutrophils (Brinkmann et al., 2004) with a range of methods suggested to be used for its assessment, including flow cytometry and ELISA-based methods, but with microscopy-based approaches still remaining the most specific and informative (Stoimenou et al., 2022).

Low-grade chronic inflammation is suggested to contribute to sarcopenia development. It was found that patients with sarcopenia have increased pro-inflammatory cytokine levels (interleukin-6, tumor necrosis factor α (TNFα)), C-reactive protein, and decreased levels of anti-inflammatory cytokine interleukin-10 (Wilson et al., 2017; Wang, 2022). In age-related sarcopenia, the dysregulation of neutrophil chemotaxis may play a key role in secondary systemic inflammation. The underlying mechanism may be dysregulated phosphoinositide 3-kinase (PI3K) activity. As a result, neutrophils migrate with less accuracy, which potentially causes increased collateral damage to muscle tissue (Sapey et al., 2014). We found a significantly elevated number of NET-forming neutrophils in the absence of any stimulus in patients with sarcopenia without an underlying cause. NET formation is known to capture, neutralize, and eliminate bacteria (Brinkmann et al., 2004), fungi (Urban et al., 2006), viruses (Saitoh et al., 2012), and even parasites (Abi Abdallah et al., 2012). However, NET components can potentially be deleterious and can also induce tissue damage, contributing to the pathogenesis of various diseases (e.g., rheumatoid arthritis, systemic lupus erythematosus, idiopathic inflammatory myopathies (Wigerblad and Kaplan, 2023), and systemic inflammation). This suggests possible involvement of NET formation in the pathogenesis of low-grade systemic inflammation and collateral muscle damage in primary sarcopenia.

On the contrary, the majority of cirrhotic patients within our study, either with or without sarcopenia, had spontaneous NET formation levels comparable to those in healthy controls. The prevalence of sarcopenia in liver diseases ranges from 30 to 70 percent (Dasarathy and Merli, 2016), and sarcopenia is associated with an increased mortality rate in cirrhotic patients (Tantai et al., 2022), which indicates the importance of studying the pathogenesis of sarcopenia in cirrhosis. There are only a few papers describing NET formation in cirrhotic patients. The plasma of patients with decompensated cirrhosis was shown to induce NET formation in isolated neutrophils from healthy controls (Sehgal et al., 2022), and levels of H3Cit-DNA, myeloperoxidase (MPO)-DNA, and cell-free DNA (markers of NET formation) were shown to be significantly elevated in patients with cirrhosis compared to healthy controls, which was associated with cirrhosis severity (Blasi et al., 2019; Zenlander et al., 2021). However, in these studies, NET formation was measured either in healthy control neutrophils in response to patients’ plasma or plasma markers of NET formation were measured and, therefore, provide only indirect evidence of possibly elevated spontaneous NET formation in patients with cirrhosis, which makes it difficult to compare their results to our findings, as we measured NET formation ability directly in freshly isolated neutrophils from cirrhotic patients. Another study described elevated spontaneous NET formation assessed in whole blood from cirrhotic patients stained with FITC-MPO antibodies and SYTOX red and analyzed by flow cytometry (Wu et al., 2021). The discrepancies in our study can be explained by different methodologies and incubation times used, which is an inherent limitation of non-standardized immunology methods. However, we also observed a highly elevated level of spontaneous NET formation in a small fraction of cirrhotic patients with or without sarcopenia. The fact that in the group of patients with both cirrhosis and sarcopenia there was also no significant increase in spontaneous NET formation suggests that either cirrhosis-associated immunosuppression might suppress sarcopenia-associated increase in NET formation or that the immune function changes and pathogenesis of primary and cirrhosis-associated sarcopenia are different.

Furthermore, we found suppressed NET formation in response to PMA and no change in the NET formation level in response to *E.coli* in patients with cirrhosis with or without sarcopenia and patients with primary sarcopenia compared to healthy controls. One study showed that NET formation in response to PMA is decreased in cirrhotic patients compared to healthy controls (Agraz-Cibrian et al., 2016; Agraz-Cibrian et al., 2018). However, another study showed an elevated NET formation level in response to PMA and *E.coli* in patients with cirrhosis compared to healthy controls (Wu et al., 2021). These discrepancies might be explained by differences in the methodologies used to assess NET formation, as well as the differences in patients’ characteristics regarding the severity and etiology of cirrhosis. The decrease in NET formation in response to one of the strongest synthetic neutrophil stimuli can be explained by neutrophil exhaustion due to chronic low-grade inflammation. The fact that NET formation in response to *E.coli* is intact can indicate that the ability of neutrophils to mount an adequate NET formation response against bacterial pathogens is preserved; therefore, NET formation changes rather do not directly contribute to the increased bacterial infections rate in these patients.

The biggest limitation of microscopy-based methods though is that they are laborious and time-consuming, which makes it complicated to apply them in clinical studies. Post-assay microscopy picture analysis and quantification of NET formation is one of the most laborious and prone to observer bias parts of these methods. To overcome this issue, several semi-automated and automated approaches were suggested in the recent years (Henneck et al., 2023). We decided to use the DANA algorithm (Rebernick et al., 2018), which is freely available and is easy to customize, optimize, implement, and control the quality of NET identification, which allows rapid quantification of NET formation percentage not only per image, but per sample, as it can automatically summarize the data from all the images taken per condition per patient, it requires only a single fluorescent channel (DNA staining), which is easier to implement within clinical studies as it is much cheaper and less time-consuming than two-channel staining (including staining for one of the NET markers, like neutrophil elastase or citrullinated histone H3 (H3Cit), apart from DNA staining). We successfully optimized this semi-automated image analysis method for our data and validated on a small dataset to make sure that it does not produce any systematic error compared to counting by eye. The results were in line with the original publication of the DANA algorithm (Rebernick et al., 2018) and revealed that image analysis by DANA was comparable to counting by eye in most of the samples analyzed. To further increase the specificity of the approach and to eliminate possible NET formation misidentification, which might occur in some of the images, we added a supervised quality control step to the analysis workflow, when the outlier NET formation values identified by DANA were also re-counted by eye, and only then the decision was made whether to accept the DANA quantified value or not. Despite this additional step, the average time of NET formation analysis per image was reduced six times compared to counting by eye, which makes this method of NET formation analysis a reliable, standardized, and fast alternative to assessment of microscopy-based NET formation assays assessed by eye. As a result, we quantified NET formation in microscopy images from 92 patients and 10 healthy controls, which is according to our knowledge, to date, the largest clinical study which assessed NET formation using the microscopy-based method.

Bacterial infections are highly prevalent among patients with cirrhosis and can worsen their prognosis by increasing the mortality rate four times (Tantai et al., 2022). Cirrhosis-associated immune dysfunction (CAID) is characterized by both systemic inflammation and immune deficiency and greatly contributes to the high bacterial infection rate. Most of anti-pathogen functions of circulating neutrophils were described to be impaired, including phagocytosis, chemotaxis, ROS production, degranulation, and bacterial killing. Phagocytic capacity is usually found to be decreased and number of non-phagocytic cells increased in patients with liver cirrhosis. As for ROS production, the number of neutrophils with basal ROS production (or resting burst) and ROS production in response to fMLF (reflecting the number of primed neutrophils) was found to be elevated, and the number of neutrophils with ROS production in response to *E. coli* was found to be either decreased or unchanged in patients with liver cirrhosis compared to healthy controls (Balazs and Stadlbauer, 2023). We did not find significant changes in either phagocytosis or ROS production of neutrophils in cirrhotic patients with or without sarcopenia in this study. This might be explained by the limited sample size to detect statistically significant differences, which might be a limitation of our study, or by the high number of patients with compensated cirrhosis, where the changes in neutrophil function are not that prominent. Some patients from the group of cirrhotic patients without sarcopenia though had dysfunctional phagocytosis. Patients with only sarcopenia also did not show any significant differences in neutrophil phagocytosis and ROS production, suggesting that these neutrophil functions might not be affected and might not play a role in the pathogenesis of primary sarcopenia. We might also speculate that spontaneous NET formation, which we observed in neutrophils from patients with primary sarcopenia, is facilitated by ROS-independent mechanisms as we did not detect any changes in neutrophil ROS production in these patients. The significantly higher number of neutrophils with ROS production in response to fMLF in healthy controls than in patients’ groups comprised approximately 3% of neutrophils and might indicate that neutrophils of patients are exhausted due to excessive priming and are not able to mount the response toward fMLF, which is comparable to healthy controls.

In conclusion, we successfully used a semi-automated NET formation quantification method, which simplifies the application of microscopy-based NET formation assessment methods in clinical studies. We found that circulating neutrophils from patients with primary sarcopenia are prone to spontaneous NET formation, which might contribute to primary sarcopenia pathogenesis. However, neither NET formation or neutrophil phagocytosis in response to *E.coli* nor the number of neutrophils undergoing ROS production were affected in patients with sarcopenia, suggesting the intact neutrophil anti-pathogen function, despite neutrophil over-activation. Interestingly, patients with cirrhosis-associated sarcopenia did not have the same neutrophil phenotype, arguing that there are differences in the pathogenesis and course of cirrhosis-associated sarcopenia compared to primary sarcopenia. We did not reveal prominent changes in NET formation in patients with cirrhosis in this study, apart from the significant decrease in NET formation response to PMA.

Future studies should aim to further explore NET formation potential as a biomarker of sarcopenia and to decipher the mechanism of spontaneous NET formation in primary sarcopenia and its involvement in sarcopenia pathogenesis, as well as explore more differences in the role of neutrophils depending on the etiology of sarcopenia. Furthermore, the potential of NET formation targeting therapeutic approaches may be studied regarding sarcopenia prevention and treatment as they have been suggested for other diseases associated with spontaneous NET formation (Honda and Kubes, 2018; van der Windt et al., 2018). Further development of NET formation assessment methodologies is also needed to be able to implement NET formation assessment as a standard lab parameter, simplifying its use as a biomarker and diagnostic criteria of different diseases.

## Data Availability

The raw data supporting the conclusion of this article will be made available by the authors, without undue reservation.

## Abbreviations

ALT, alanine transaminase; AST, aspartate transaminase; AP, alkaline phosphatase; CRP, C-reactive protein; GGT, gamma‐glutamyl transferase; HCT, hematocrit; INR, international normalized ratio; NET, neutrophil extracellular trap; DANA, (DNA Area and NETosis Analysis); PMA, phorbol 12-myristate 13-acetate; DAPI, 4′,6-diamidino-2-phenylindole; RPMI, Roswell Park Memorial Institute; ROS, reactive oxygen species; HCC, hepatocellular carcinoma; fMLF, N-formylmethionine-leucyl-phenylalanine; BMI, body mass index; MELD, the model for end-stage liver disease; HCV, hepatitis C virus; NASH, non-alcoholic steatohepatitis; ELISA, the enzyme-linked immunosorbent assay; ROI, region of interest; TNFα, tumor necrosis factor α; H3Cit, citrullinated histone H3; MPO, myeloperoxidase; HCC, hepatocellular carcinoma.

## References

[B1] Abi Abdallah D. S. Lin C. Ball C. J. King M. R. Duhamel G. E. Denkers E. Y. (2012). Toxoplasma gondii triggers release of human and mouse neutrophil extracellular traps. Infect. Immun. 80 (2), 768–777. 10.1128/IAI.05730-11 22104111 PMC3264325

[B2] Agraz-Cibrian J. M. Delgado-Rizo V. Segura-Ortega J. E. Maldonado-Gomez H. A. Zambrano-Zaragoza J. F. Duran-Avelar M. J. (2018). Impaired neutrophil extracellular traps and inflammatory responses in the peritoneal fluid of patients with liver cirrhosis. Scand. J. Immunol. 88 (5), e12714. 10.1111/sji.12714 30226638

[B3] Agraz-Cibrian J. M. Segura-Ortega J. E. Delgado-Rizo V. Fafutis-Morris M. (2016). Alterations in neutrophil extracellular traps is associated with the degree of decompensation of liver cirrhosis. J. Infect. Dev. Ctries. 10 (5), 512–517. 10.3855/jidc.7165 27249527

[B4] Anker S. D. Morley J. E. von Haehling S. (2016). Welcome to the ICD-10 code for sarcopenia. J. Cachexia Sarcopenia Muscle 7 (5), 512–514. 10.1002/jcsm.12147 27891296 PMC5114626

[B5] Balazs I. Stadlbauer V. (2023). Circulating neutrophil anti-pathogen dysfunction in cirrhosis. JHEP Rep. 5 (11), 100871. 10.1016/j.jhepr.2023.100871 37822786 PMC10562928

[B6] Blasi A. Patel V. C. Adelmeijer J. Azarian S. Aziz F. Fernandez J. (2019). Plasma levels of circulating DNA are associated with outcome, but not with activation of coagulation in decompensated cirrhosis and ACLF. JHEP Rep. 1 (3), 179–187. 10.1016/j.jhepr.2019.06.002 32039368 PMC7001554

[B7] Brinkmann V. Laube B. Abu Abed U. Goosmann C. Zychlinsky A. (2010). Neutrophil extracellular traps: how to generate and visualize them. J. Vis. Exp. 36, 1724. 10.3791/1724 PMC3125121 20182410

[B8] Brinkmann V. Reichard U. Goosmann C. Fauler B. Uhlemann Y. Weiss D. S. (2004). Neutrophil extracellular traps kill bacteria. Science 303 (5663), 1532–1535. 10.1126/science.1092385 15001782

[B9] Cruz-Jentoft A. J. Bahat G. Bauer J. Boirie Y. Bruyere O. Cederholm T. (2019). Sarcopenia: revised European consensus on definition and diagnosis. Age Ageing 48 (1), 601–631. 10.1093/ageing/afz046 PMC6593317 31081853

[B10] Cruz-Jentoft A. J. Sayer A. A. (2019). Sarcopenia. Lancet. 393 (10191), 2636–2646. 10.1016/S0140-6736(19)31138-9 31171417

[B11] Dasarathy S. Merli M. (2016). Sarcopenia from mechanism to diagnosis and treatment in liver disease. J. Hepatol. 65 (6), 1232–1244. 10.1016/j.jhep.2016.07.040 27515775 PMC5116259

[B12] Henneck T. Kruger C. Nerlich A. Langer M. Fingerhut L. Bonilla M. C. (2023). Comparison of NET quantification methods based on immunofluorescence microscopy: hand-counting, semi-automated and automated evaluations. Heliyon 9 (6), e16982. 10.1016/j.heliyon.2023.e16982 37484269 PMC10361044

[B13] Herrero-Cervera A. Soehnlein O. Kenne E. (2022). Neutrophils in chronic inflammatory diseases. Cell Mol. Immunol. 19 (2), 177–191. 10.1038/s41423-021-00832-3 35039631 PMC8803838

[B14] Honda M. Kubes P. (2018). Neutrophils and neutrophil extracellular traps in the liver and gastrointestinal system. Nat. Rev. Gastroenterol. Hepatol. 15 (4), 206–221. 10.1038/nrgastro.2017.183 29382950

[B15] Horvath A. Leber B. Schmerboeck B. Tawdrous M. Zettel G. Hartl A. (2016). Randomised clinical trial: the effects of a multispecies probiotic vs. placebo on innate immune function, bacterial translocation and gut permeability in patients with cirrhosis Aliment. Pharmacol. Ther. 44 (9), 926–935. 10.1111/apt.13788 27593544 PMC5053220

[B16] Ley K. Hoffman H. M. Kubes P. Cassatella M. A. Zychlinsky A. Hedrick C. C. (2018). Neutrophils: new insights and open questions. Sci. Immunol. 3 (30), eaat4579. 10.1126/sciimmunol.aat4579 30530726

[B17] Papayannopoulos V. (2018). Neutrophil extracellular traps in immunity and disease. Nat. Rev. Immunol. 18 (2), 134–147. 10.1038/nri.2017.105 28990587

[B18] Rebernick R. Fahmy L. Glover C. Bawadekar M. Shim D. Holmes C. L. (2018). DNA area and NETosis analysis (DANA): a high-throughput method to quantify neutrophil extracellular traps in fluorescent microscope images. Biol. Proced. Online 20, 7. 10.1186/s12575-018-0072-y 29618953 PMC5878938

[B19] Riuzzi F. Sorci G. Arcuri C. Giambanco I. Bellezza I. Minelli A. (2018). Cellular and molecular mechanisms of sarcopenia: the S100B perspective. J. Cachexia Sarcopenia Muscle 9 (7), 1255–1268. 10.1002/jcsm.12363 30499235 PMC6351675

[B20] Saitoh T. Komano J. Saitoh Y. Misawa T. Takahama M. Kozaki T. (2012). Neutrophil extracellular traps mediate a host defense response to human immunodeficiency virus-1. Cell Host Microbe 12 (1), 109–116. 10.1016/j.chom.2012.05.015 22817992

[B21] Sapey E. Greenwood H. Walton G. Mann E. Love A. Aaronson N. (2014). Phosphoinositide 3-kinase inhibition restores neutrophil accuracy in the elderly: toward targeted treatments for immunosenescence. Blood 123 (2), 239–248. 10.1182/blood-2013-08-519520 24191150 PMC3888290

[B22] Sehgal R. Kaur N. Maiwall R. Ramakrishna G. Maras J. S. Trehanpati N. (2022). Plasma proteomic analysis identified proteins associated with faulty neutrophils functionality in decompensated cirrhosis patients with sepsis. Cells 11 (11), 1745. 10.3390/cells11111745 35681439 PMC9179303

[B23] Stoimenou M. Tzoros G. Skendros P. Chrysanthopoulou A. (2022). Methods for the assessment of NET formation: from neutrophil Biology to translational research. Int. J. Mol. Sci. 23 (24), 15823. 10.3390/ijms232415823 36555464 PMC9781911

[B24] Tantai X. Liu Y. Yeo Y. H. Praktiknjo M. Mauro E. Hamaguchi Y. (2022). Effect of sarcopenia on survival in patients with cirrhosis: a meta-analysis. J. Hepatol. 76 (3), 588–599. 10.1016/j.jhep.2021.11.006 34785325

[B25] Urban C. F. Reichard U. Brinkmann V. Zychlinsky A. (2006). Neutrophil extracellular traps capture and kill Candida albicans yeast and hyphal forms. Cell Microbiol. 8 (4), 668–676. 10.1111/j.1462-5822.2005.00659.x 16548892

[B26] van der Windt D. J. Sud V. Zhang H. Varley P. R. Goswami J. Yazdani H. O. (2018). Neutrophil extracellular traps promote inflammation and development of hepatocellular carcinoma in nonalcoholic steatohepatitis. Hepatology 68 (4), 1347–1360. 10.1002/hep.29914 29631332 PMC6173613

[B27] Wang T. (2022). Searching for the link between inflammaging and sarcopenia. Ageing Res. Rev. 77, 101611. 10.1016/j.arr.2022.101611 35307560

[B28] Wigerblad G. Kaplan M. J. (2023). Neutrophil extracellular traps in systemic autoimmune and autoinflammatory diseases. Nat. Rev. Immunol. 23 (5), 274–288. 10.1038/s41577-022-00787-0 36257987 PMC9579530

[B29] Wilson D. Jackson T. Sapey E. Lord J. M. (2017). Frailty and sarcopenia: the potential role of an aged immune system. Ageing Res. Rev. 36, 1–10. 10.1016/j.arr.2017.01.006 28223244

[B30] Wu W. Sun S. Wang Y. Zhao R. Ren H. Li Z. (2021). Circulating neutrophil dysfunction in HBV-related acute-on-chronic liver failure. Front. Immunol. 12, 620365. 10.3389/fimmu.2021.620365 33717119 PMC7947208

[B31] Zenlander R. Havervall S. Magnusson M. Engstrand J. Agren A. Thalin C. (2021). Neutrophil extracellular traps in patients with liver cirrhosis and hepatocellular carcinoma. Sci. Rep. 11 (1), 18025. 10.1038/s41598-021-97233-3 34504150 PMC8429678

