# Supplementary material for: Primary sarcopenia is associated with elevated spontaneous NET formation
Source: Front Cell Dev Biol. 2024 Mar 5;12:1347495. doi: 10.3389/fcell.2024.1347495 (PMC10948394; doi:10.3389/fcell.2024.1347495)
Supplement: Supplementary file 1 [file DataSheet1.pdf]

Supplementary material

Primary sarcopenia is associated with elevated spontaneous NET formation

Irina Balazs[1,2], Manuel Stelzer[1], Julia Traub[3], Angela Horvath[1,2], Nicole Feldbacher[1,2] and Vanessa Stadlbauer[1,2]*

[1]Department of Internal Medicine, Division of Gastroenterology and Hepatology, Medical University of Graz, Graz, Austria

[2]Center for Biomarker Research in Medicine (CBmed), Graz, Austria

[3]Department of Clinical Medical Nutrition, Medical University of Graz, Graz, Austria

* **Correspondence:**
Vanessa Stadlbauer
vanessa.stadlbauer@medunigraz.at

Table of contents:

Supplementary Fig.1

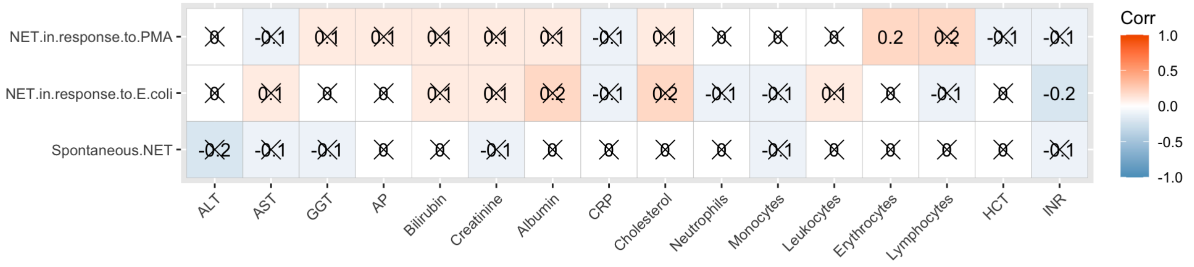

Supplementary Fig. 1. Correlation between NET formation and other clinical data of patients. Correlation matrix between NET formation parameters and other clinical parameters of patients (n=89). Spearman's correlation coefficient with Benjamini-Hochberg adjustment for multiple tests was calculated. Crossed squares mean insignificant correlation. ALT: alanine transaminase; AST: aspartate transaminase; GGT: gamma-glutamyl transferase; AP: alkaline phosphatase; CRP: C-reactive protein; HCT: hematocrit; INR: international normalized ratio.

Supplementary Fig.2

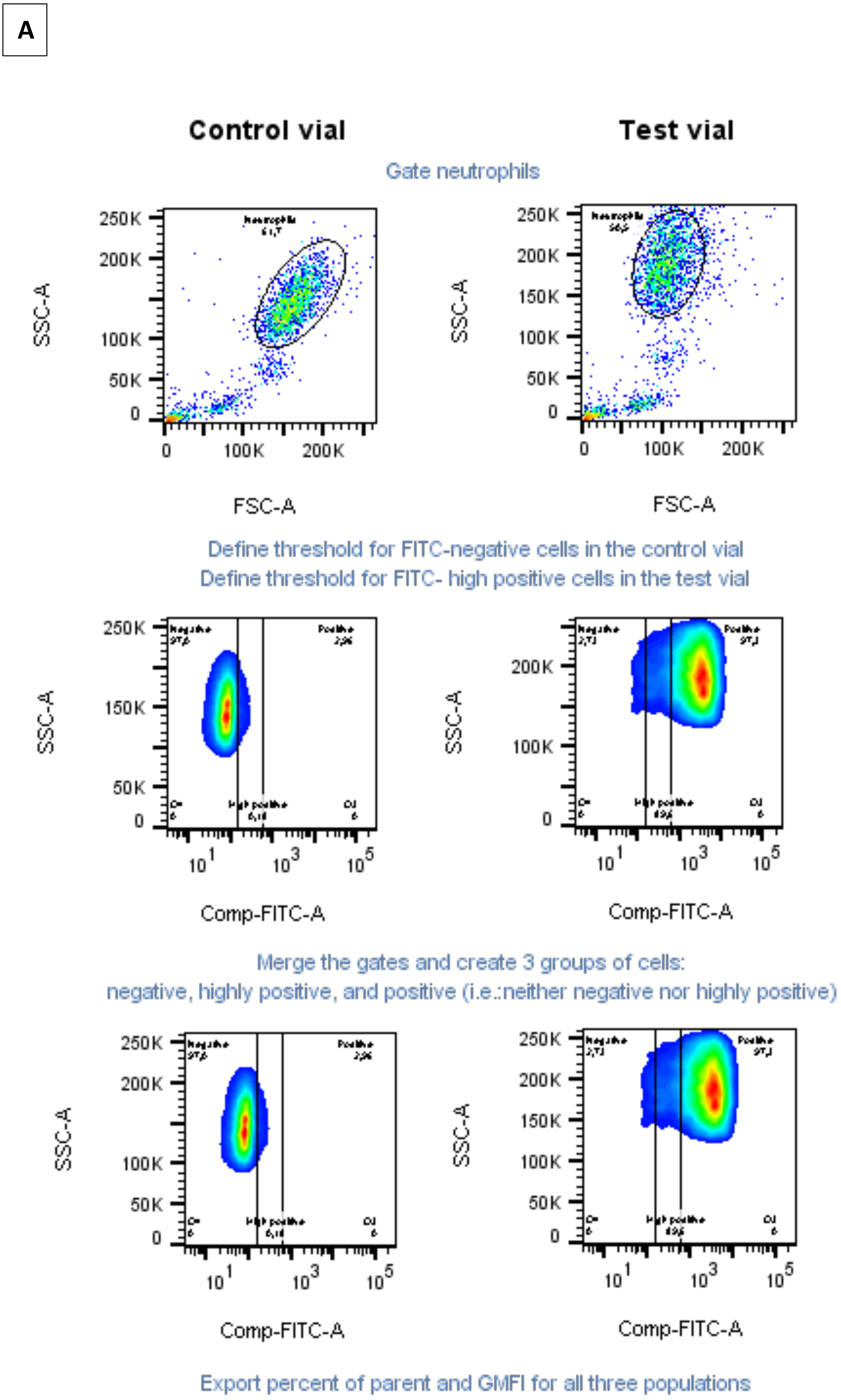
A
Control vial
Test vial
Gate neutrophils
SSC-A
FSC-A
250K
200K
150K
100K
50K
0
100K
200K
Define threshold for FITC-negative cells in the control vial
Define threshold for FITC- high positive cells in the test vial
Comp-FITC-A
Merge the gates and create 3 groups of cells:
negative, highly positive, and positive (i.e.:neither negative nor highly positive)
Export percent of parent and GMFI for all three populations

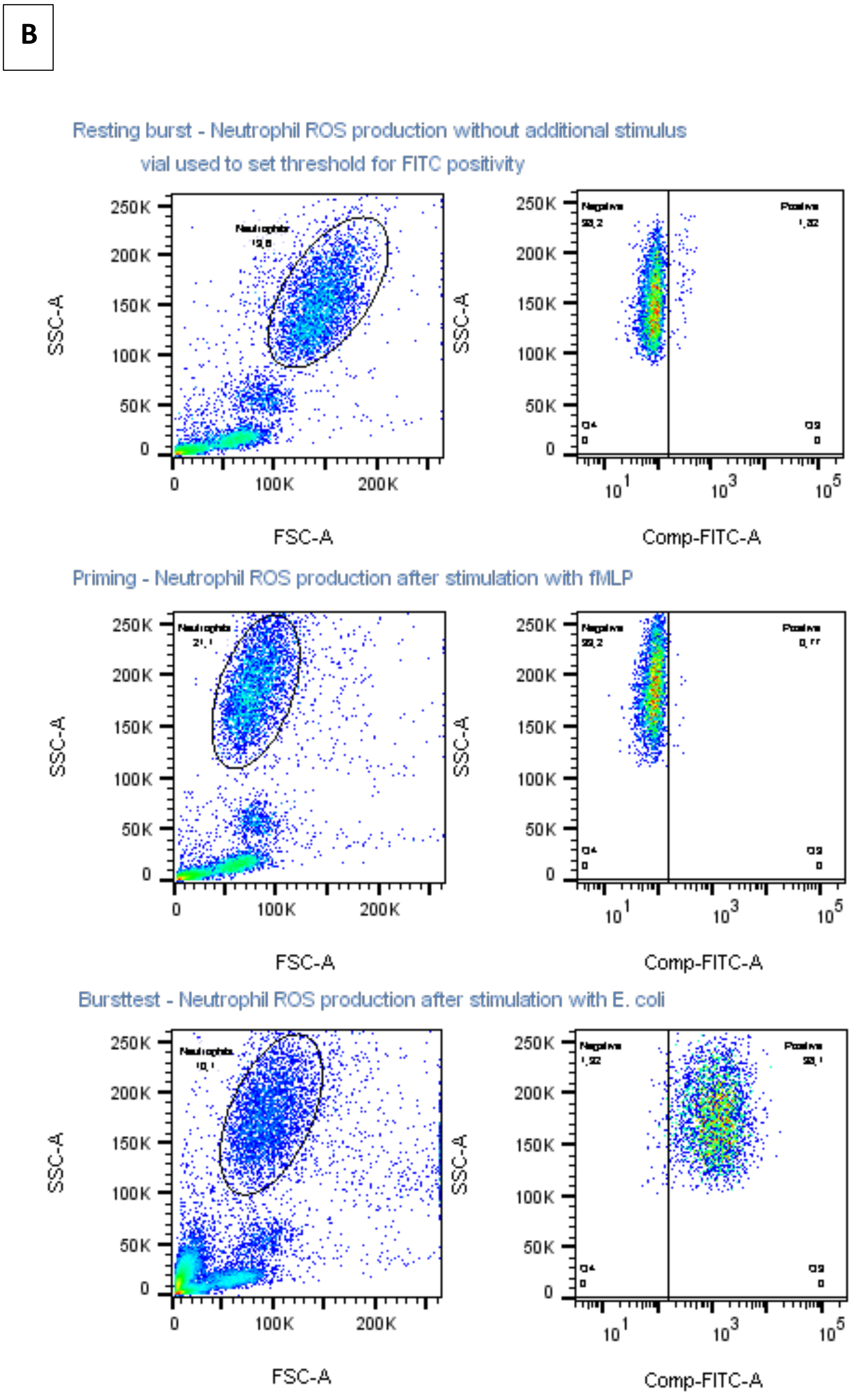

Supplementary Fig. 2 Gating strategy of flow cytometry analysis of phagocytosis and ROS production. (A) 100 ul of the whole blood was mixed with 20µl of stabilized and opsonized FITC-labelled *E. coli* suspension (2 x 10^9 bacteria/ml)

in two tubes. One tube (control) stayed on ice; another one was incubated for 10 min at 37°C. Quenching solution was added to avoid counting of the cell membrane-attached not engulfed bacteria. After washing step cells were fixed and red blood cells were lysed. LSRII flow cytometer (BD Biosciences, San Jose, California, USA) with BD FACS Diva 6.2 software (BD Bioscience, San Jose, California, USA) were used to record 10,000 neutrophils. Further analysis was performed in FlowJoTM V10 software (BD Biosciences, San Jose, California, USA) and the percentage of non-phagocytic neutrophils as well as phagocytic capacity of neutrophils were calculated. Gating algorithm in control and test tubes is presented. (B) 100µl of the whole blood was mixed with 20µl of either wash solution (resting burst), N-Formylmethionine-leucyl-phenylalanine (fMLF) (priming), phorbol myristate acetate (PMA) (positive control) or stabilized and opsonized (non-labelled) *E.coli* (1-2x10^9 bacteria/ml) (bursttest). Tubes were incubated for 10 min at 37°C. Substrate solution containing dihydrorhodamine 123 was added to all the tubes and incubated for 10 min at 37°C. Cells were fixed and red blood cells were lysed. LSRII flow cytometer (BD Bioscience, San Jose, California, USA) with BD FACS Diva 6.2 software (BD Bioscience, San Jose, California, USA) were used to record 10,000 neutrophils. Further analysis was performed in FlowJoTM V10 software (BD Biosciences, San Jose, California, USA). Gating algorithm for resting burst, priming and bursttest is presented in the figure.

Supplementary Fig.3

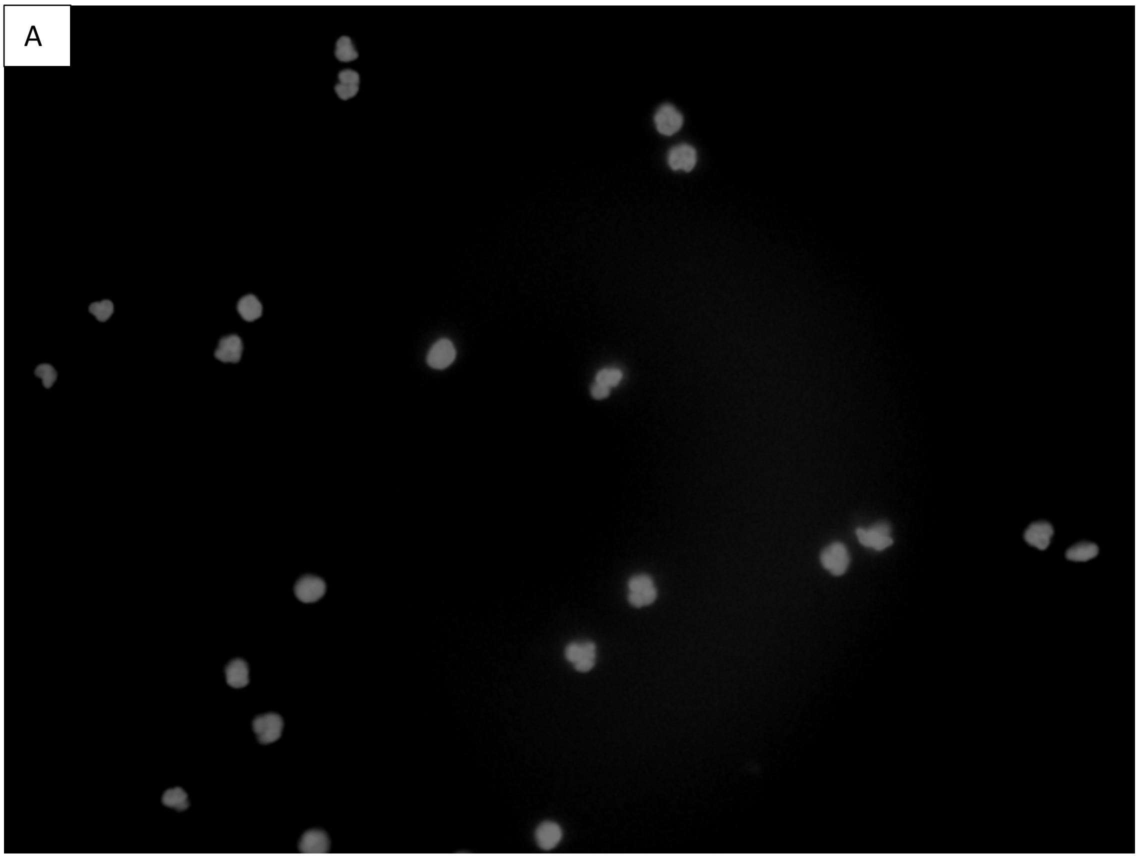

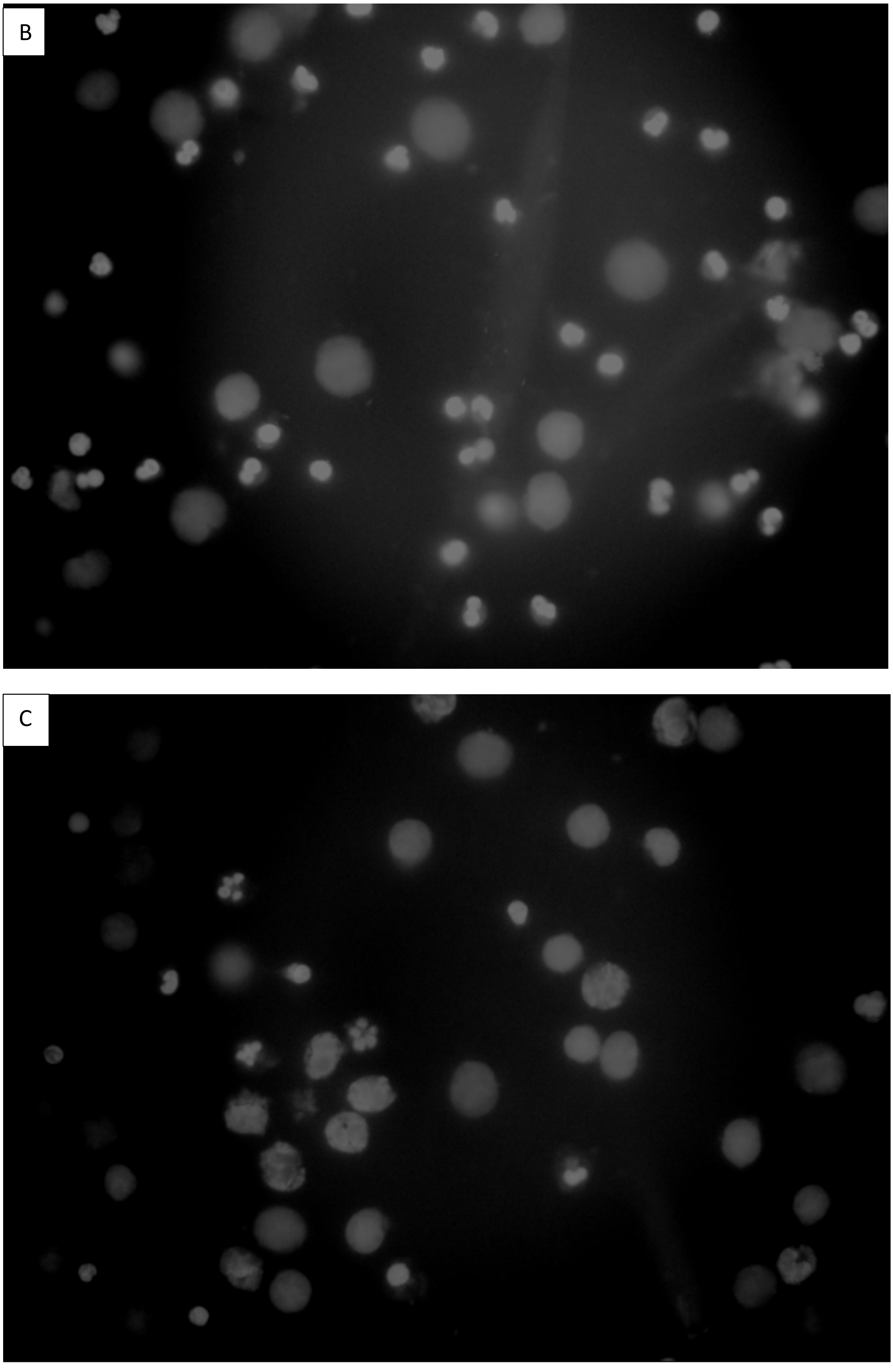
B
C

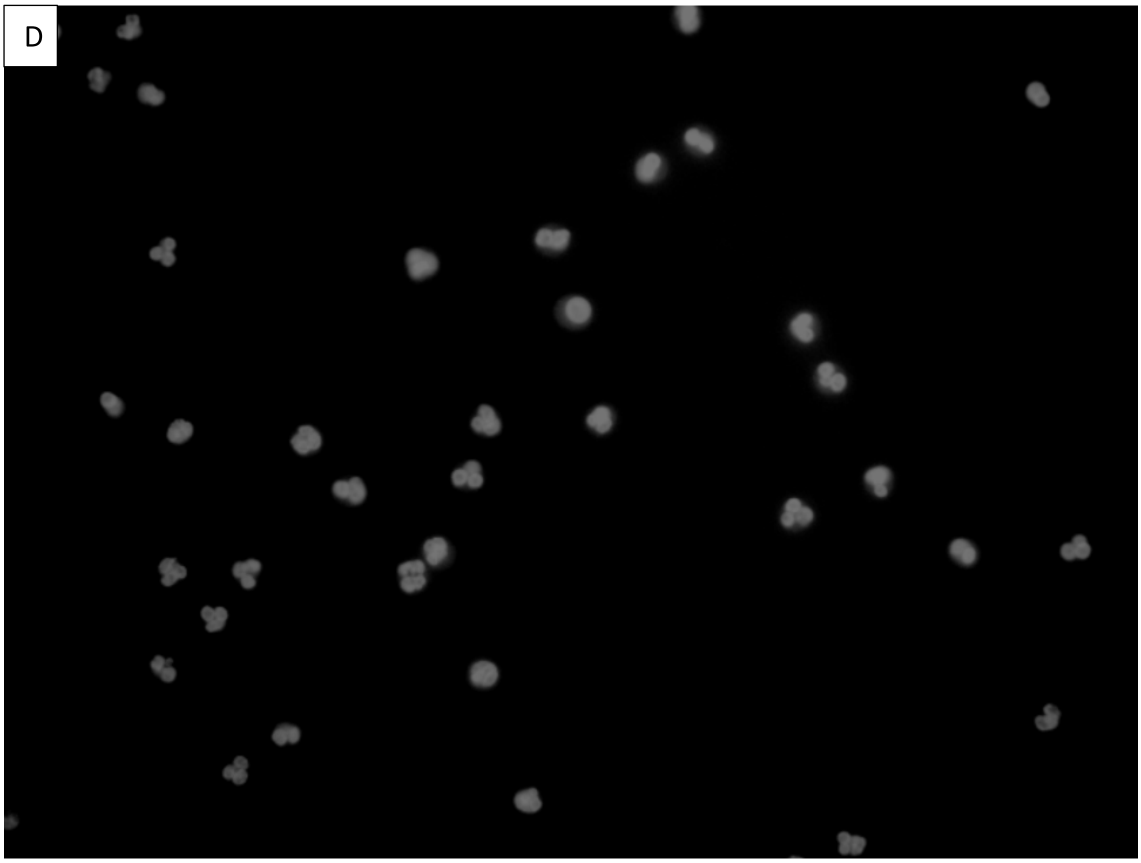
D

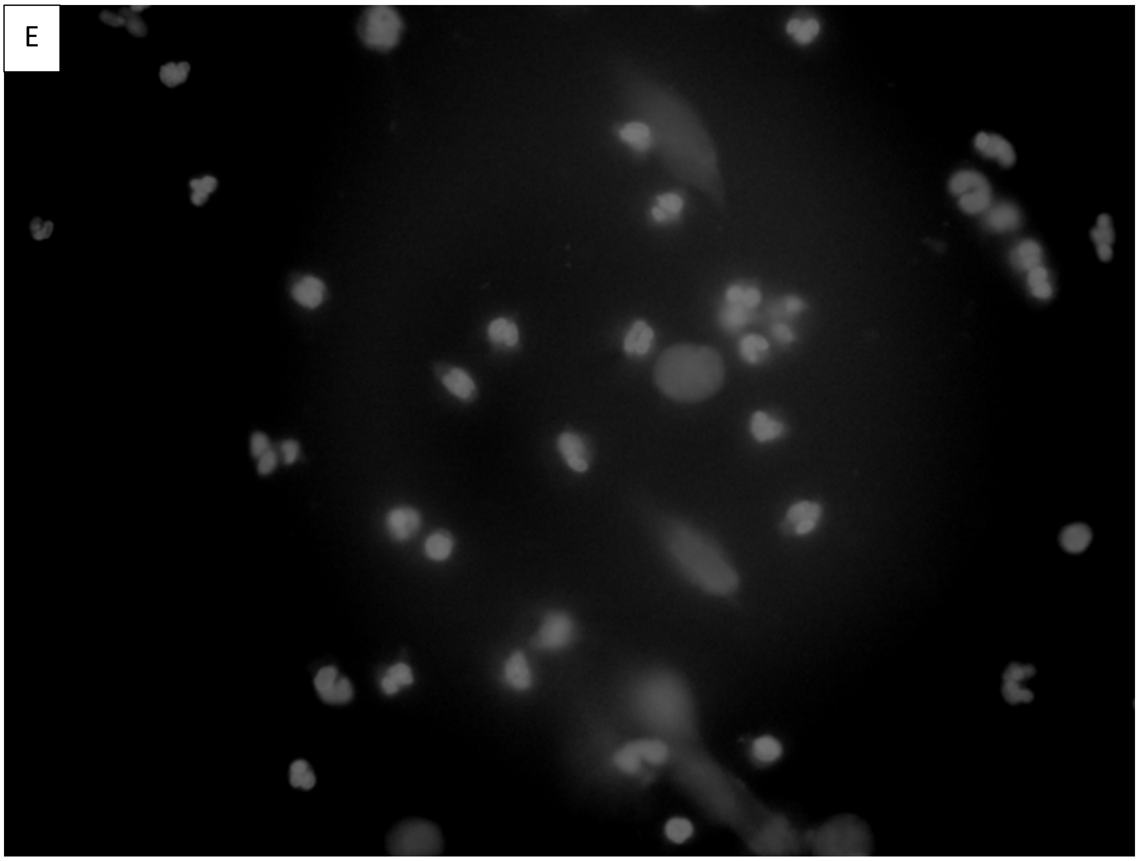
E

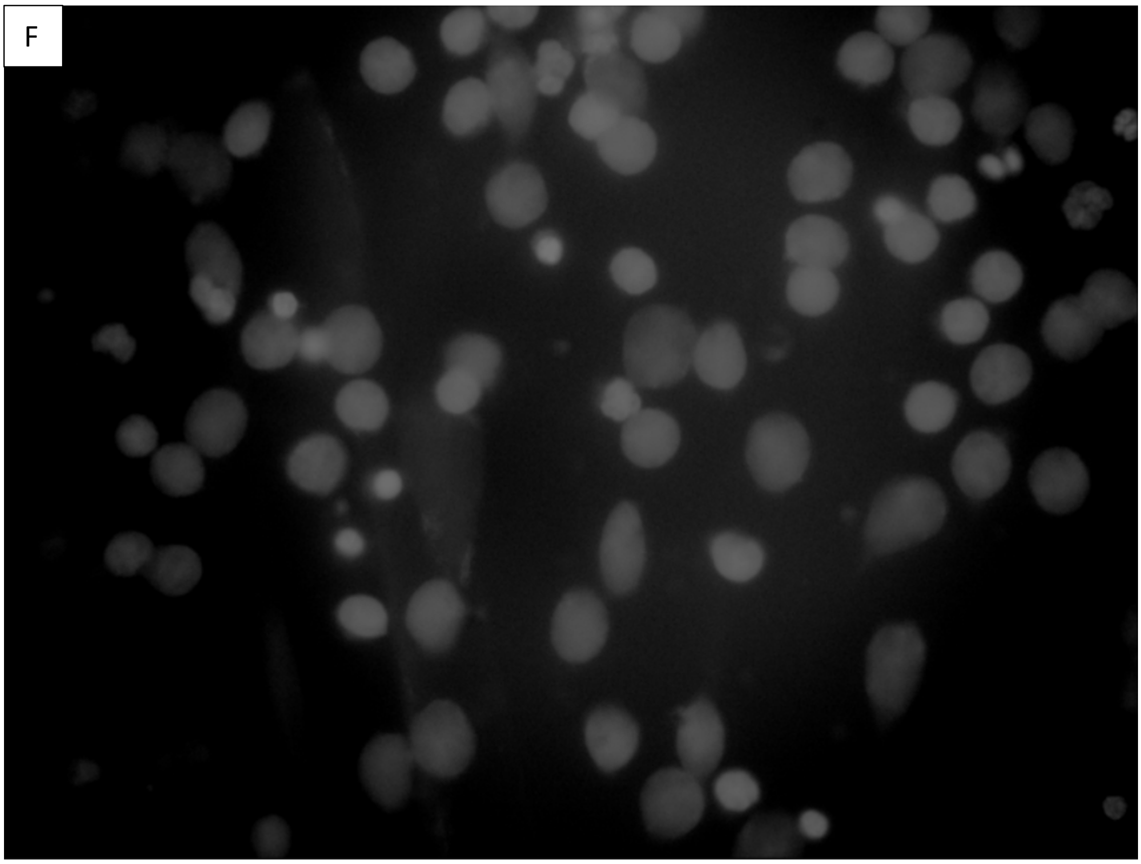
F

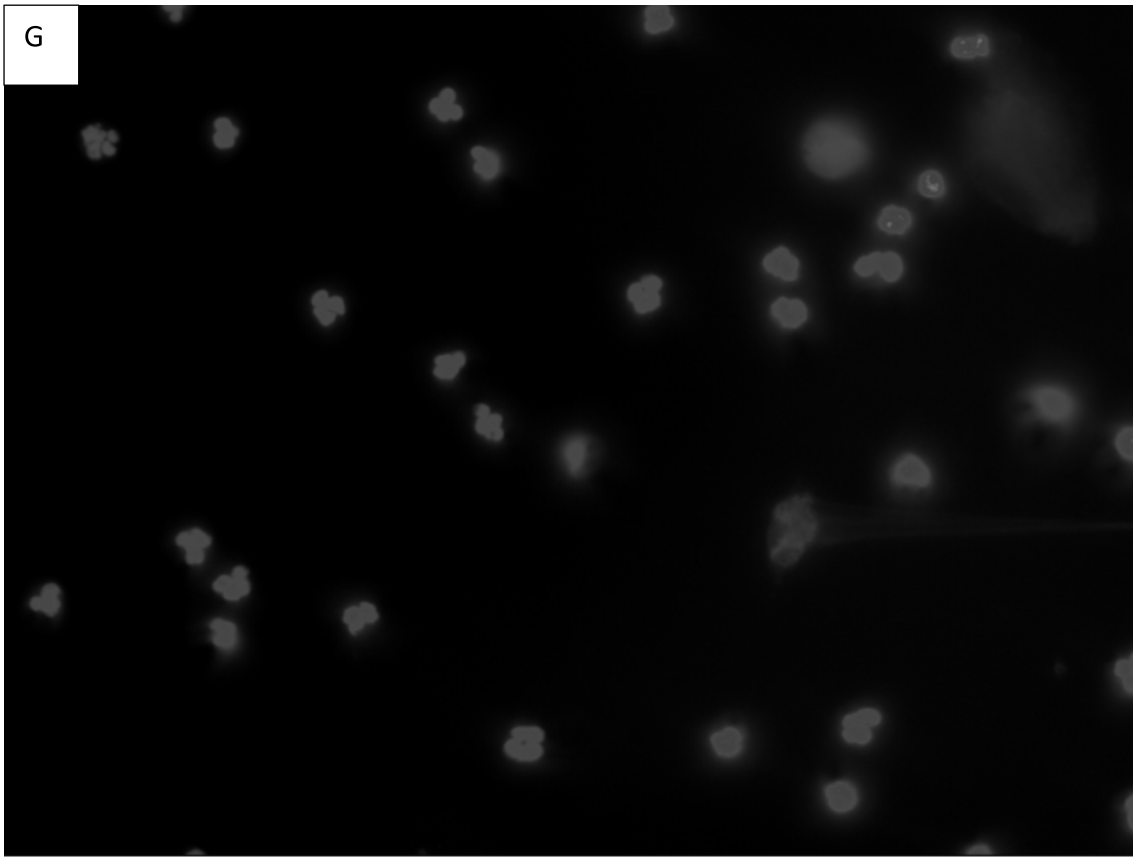
G

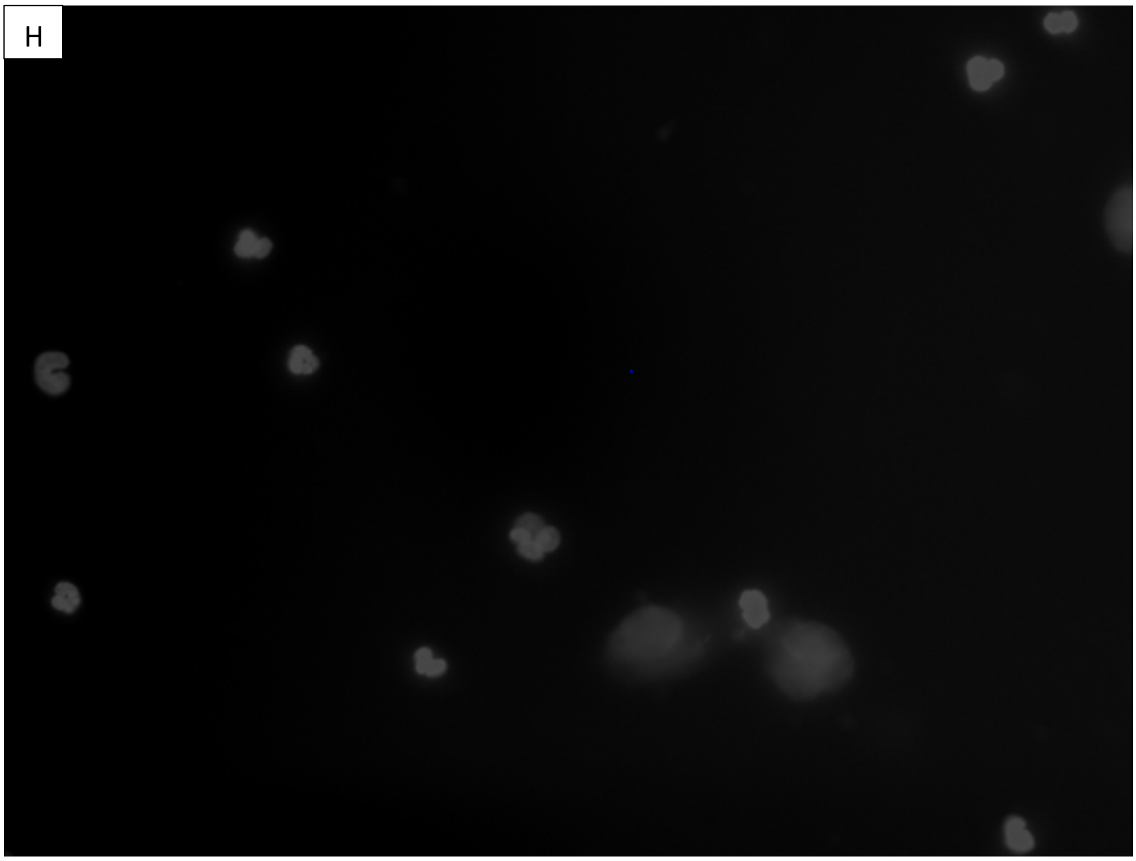
H

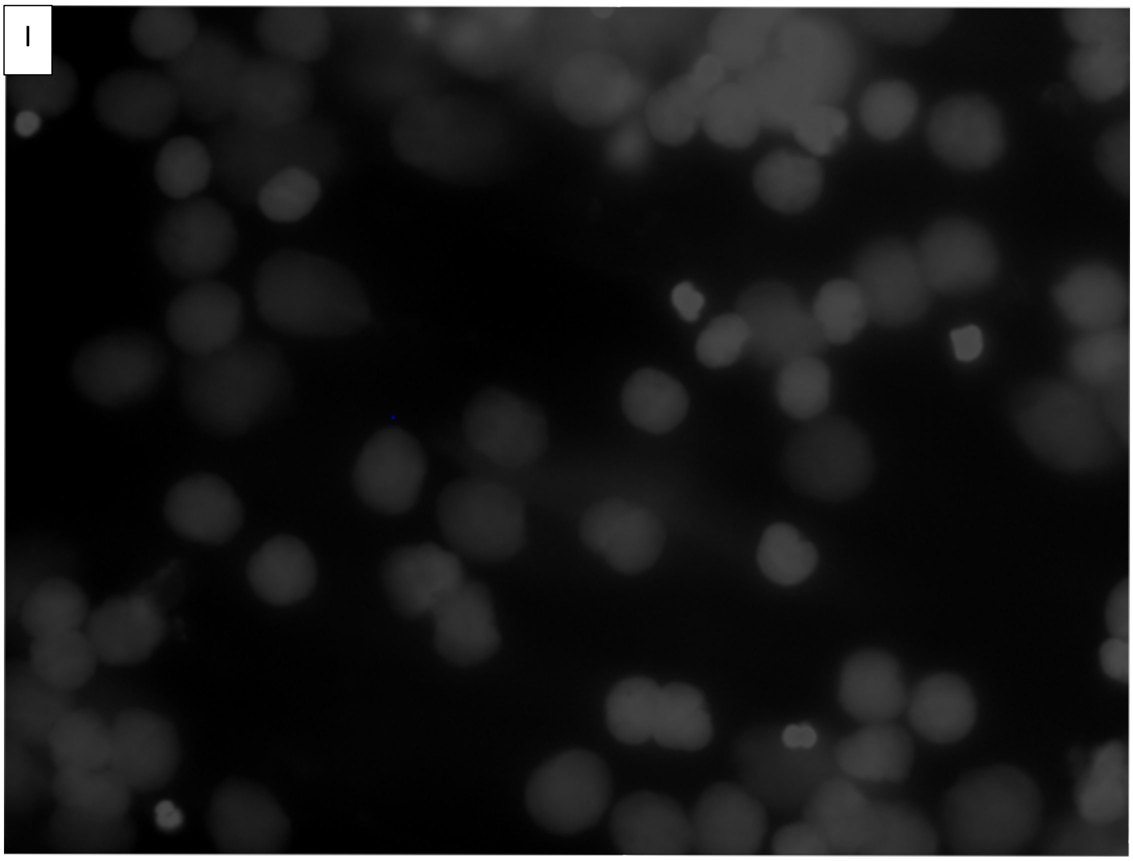
I

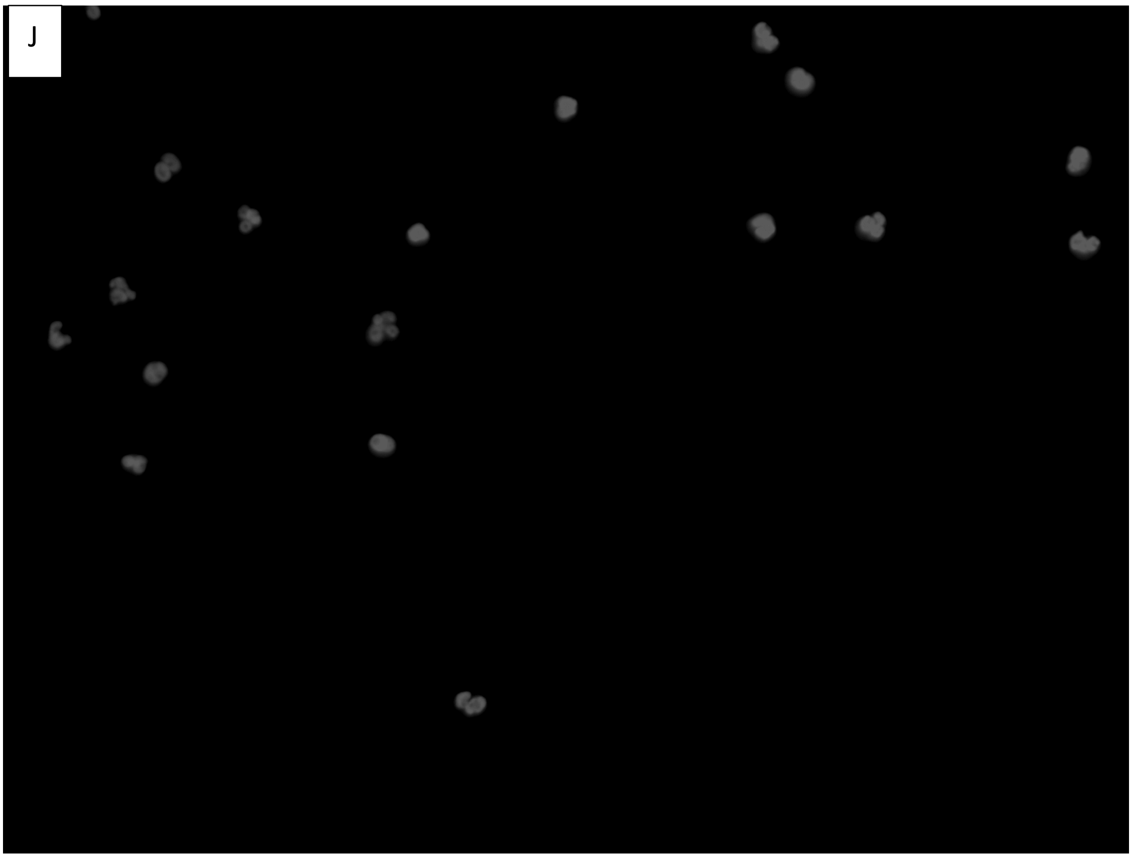
J

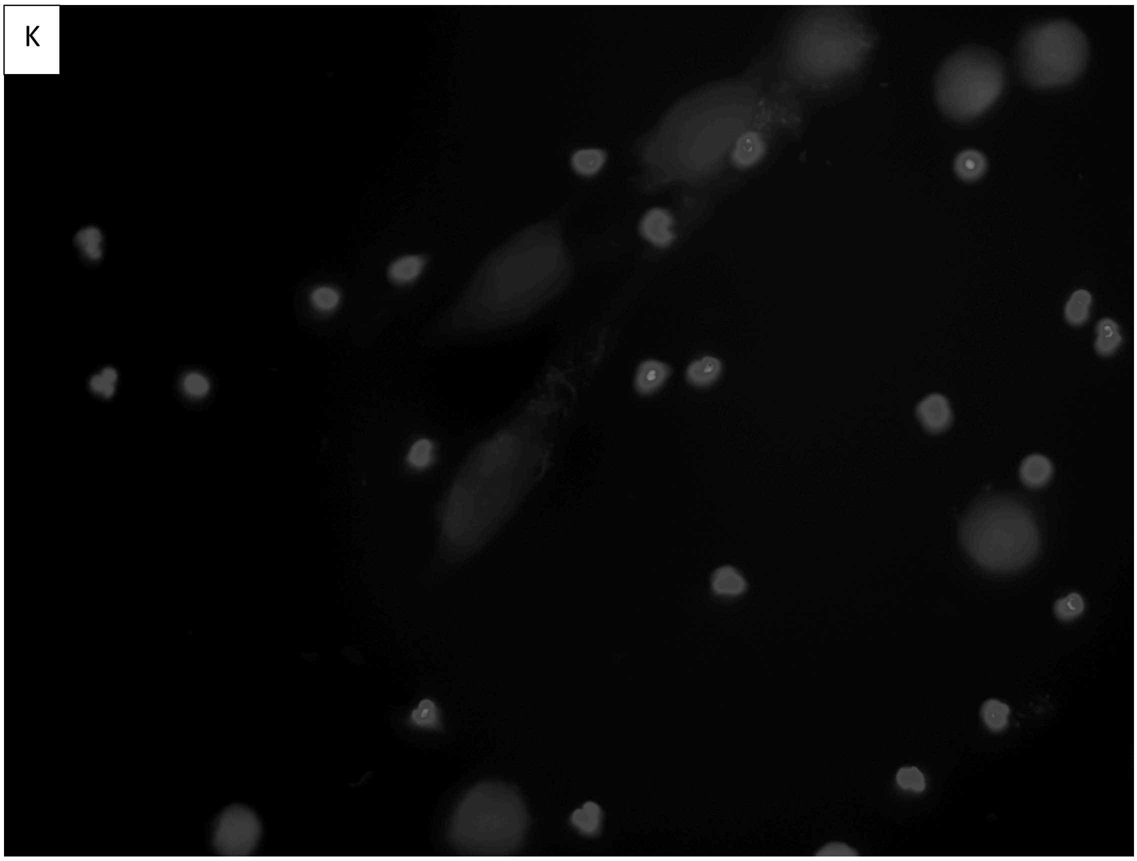
K

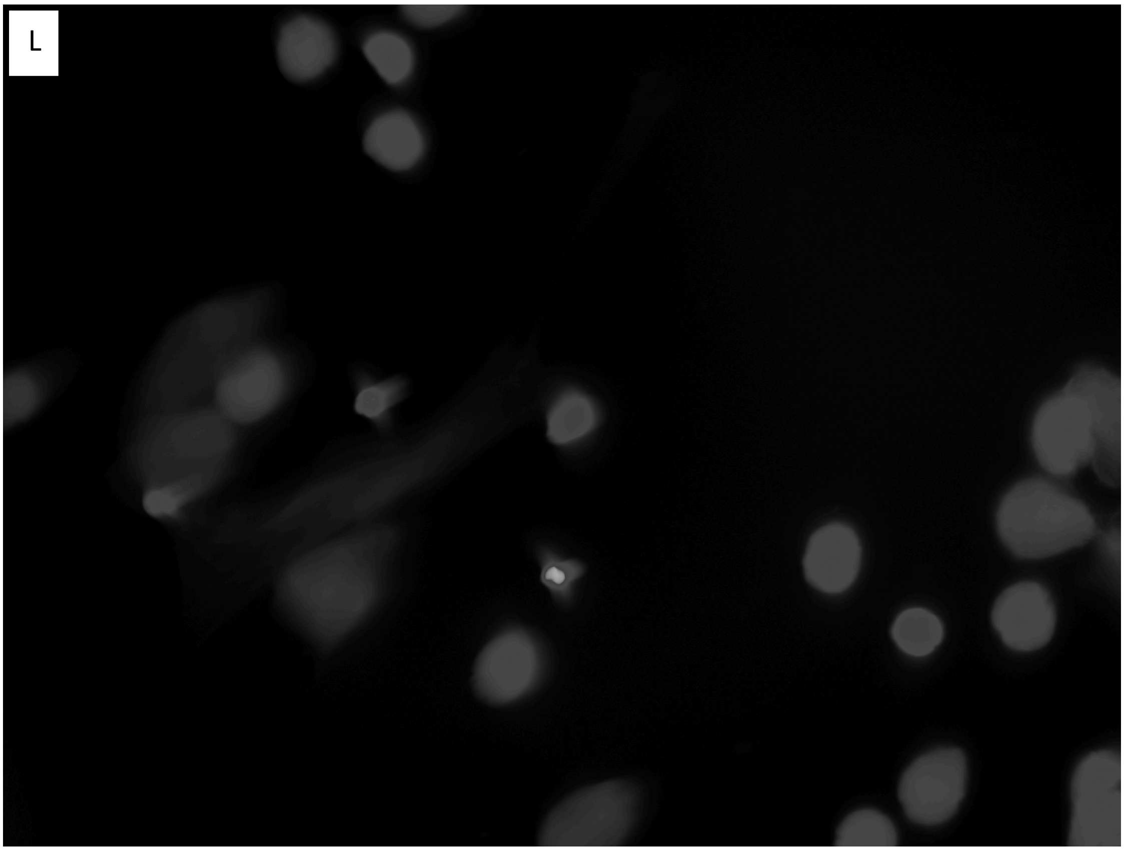

Supplementary Fig. 3 Representative pictures of NET formation in neutrophils from patients' groups and healthy controls after staining with DAPI. (A-L) Neutrophils were isolated from peripheral blood of patients with cirrhosis (A-C), cirrhosis and sarcopenia (D-F), sarcopenia (G-I) and healthy controls (J-L), NET formation was induced with RPMI (spontaneous NET formation) (A, D, G, J), *E.coli* (100ul of heat-inactivated *E. coli* BL21 $5x10^8$ CFU/ml per 500ul of $4x10^5$ neutrophils/ml) (B, E, H, K) or 100nM PMA (C, F, I, L) for 2 hours at 37°C. Cells were fixed and stained with DAPI. Pictures of 10 random fields of vision per slide were taken with Olympus BX51 Fluorescence Microscope (Olympus, Shinjuku, Tokyo, Japan) at 600x total magnification. NET: neutrophil extracellular trap; DAPI: 4′,6-diamidino-2-phenylindole; RPMI: Roswell Park Memorial Institute medium; PMA: phorbol 12-myristate 13-acetate.

Supplementary Table 1

	**Cirrhosis**	**Cirrhosis and Sarcopenia**	**Sarcopenia**
**N**	25	52	15

**PPI, yes/no, N**	13/12	25/27	5/10
**Betablockers, yes/no, N**	15/10	32/20	2/13
**Statine, yes/no, N**	6/19	4/48	2/13
**Laevolac, yes/no, N**	4/21	12/40	0/15
**Laxatives, yes/no, N**	1/24	3/12	2/13
**Diuretic, yes/no, N**	11/14	32/20	1/14
**Antihypertensive, yes/no, N**	11/14	11/41	4/11
**Antidepressants, yes/no, N**	4/21	9/43	5/10
**Opiate, yes/no, N**	2/23	4/48	3/12
**Antibiotics, yes/no, N**	3/22	6/46	0/15
**Sedative, yes/no, N**	2/23	9/43	2/13
**Immunosupressive, yes/no, N**	3/22	1/51	2/13
**Cholesterol lowering drugs, yes/no, N**	1/24	0/52	1/14
**Testosteron drugs, yes/no, N**	0/25	0/52	0/15
**Botanical drugs, yes/no, N**	1/24	2/50	0/15

PPI: proton pump inhibitors.
